# Subset binding enables detection of multimodal patient subgroup patterns and drug target discovery in idiopathic pulmonary fibrosis

**DOI:** 10.1093/bib/bbag153

**Published:** 2026-04-14

**Authors:** Yayoi Natsume-Kitatani, Mari N Itoh, Yoshito Takeda, Masataka Kuroda, Haruhiko Hirata, Kotaro Miyake, Takayuki Shiroyama, Yuya Shirai, Yoshimi Noda, Yuichi Adachi, Takatoshi Enomoto, Saori Amiya, Jun Adachi, Ryohei Narumi, Satoshi Muraoka, Takeshi Tomonaga, Sadao Kurohashi, Fei Cheng, Ribeka Tanaka, Shuntaro Yada, Eiji Aramaki, Shoko Wakamiya, Yi-An Chen, Akiko Fukagawa, Chihiro Higuchi, Yosui Nojima, Takeshi Fujiwara, Chioko Nagao, Toshihiro Takeda, Yasushi Matsumura, Kenji Mizuguchi, Atsushi Kumanogoh, Naonori Ueda

**Affiliations:** Laboratory of Bioinformatics, Artificial Intelligence Center for Health and Biomedical Research, National Institutes of Biomedical Innovation, Health and Nutrition; 3-17 Senrioka-shinmachi, Settsu, Osaka 567-0085, Japan; Institute of Advanced Medical Sciences, Tokushima University, 3-18-15 Kuramoto-cho, Tokushima, Tokushima 770-8503, Japan; Institute for Protein Research, the University of Osaka, 3-2 Yamada-oka, Suita, Osaka 565-0871, Japan; Graduate School of Pharmaceutical Sciences, the University of Osaka, 1-10 Yamada-oka, Suita, Osaka 565-0871, Japan; Laboratory of Bioinformatics, Artificial Intelligence Center for Health and Biomedical Research, National Institutes of Biomedical Innovation, Health and Nutrition; 3-17 Senrioka-shinmachi, Settsu, Osaka 567-0085, Japan; Graduate School of Pharmaceutical Sciences, the University of Osaka, 1-10 Yamada-oka, Suita, Osaka 565-0871, Japan; Department of Respiratory Medicine and Clinical Immunology, the University of Osaka Graduate School of Medicine, 2-2 Yamada-oka, Suita, Osaka 565-0871, Japan; Laboratory of Bioinformatics, Artificial Intelligence Center for Health and Biomedical Research, National Institutes of Biomedical Innovation, Health and Nutrition; 3-17 Senrioka-shinmachi, Settsu, Osaka 567-0085, Japan; Discovery Technology Laboratories, Mitsubishi Tanabe Pharma Corporation, 1000 Kamoshida-cho, Aoba-ku, Yokohama, Kanagawa 227-0033, Japan; Department of Respiratory Medicine and Clinical Immunology, the University of Osaka Graduate School of Medicine, 2-2 Yamada-oka, Suita, Osaka 565-0871, Japan; Department of Respiratory Medicine and Clinical Immunology, the University of Osaka Graduate School of Medicine, 2-2 Yamada-oka, Suita, Osaka 565-0871, Japan; Department of Respiratory Medicine and Clinical Immunology, the University of Osaka Graduate School of Medicine, 2-2 Yamada-oka, Suita, Osaka 565-0871, Japan; Department of Respiratory Medicine and Clinical Immunology, the University of Osaka Graduate School of Medicine, 2-2 Yamada-oka, Suita, Osaka 565-0871, Japan; Department of Respiratory Medicine and Clinical Immunology, the University of Osaka Graduate School of Medicine, 2-2 Yamada-oka, Suita, Osaka 565-0871, Japan; Department of Respiratory Medicine and Clinical Immunology, the University of Osaka Graduate School of Medicine, 2-2 Yamada-oka, Suita, Osaka 565-0871, Japan; Department of Respiratory Medicine and Clinical Immunology, the University of Osaka Graduate School of Medicine, 2-2 Yamada-oka, Suita, Osaka 565-0871, Japan; Department of Respiratory Medicine and Clinical Immunology, the University of Osaka Graduate School of Medicine, 2-2 Yamada-oka, Suita, Osaka 565-0871, Japan; Laboratory of Proteomics for Drug Discovery, Center for Drug Design Research, National Institutes of Biomedical Innovation, Health and Nutrition, 7-6-8 Saito-Asagi, Ibaraki, Osaka 567-0085, Japan; Laboratory of Proteome Research, National Institutes of Biomedical Innovation, Health and Nutrition, 7-6-8 Saito-Asagi, Ibaraki, Osaka 567-0085, Japan; Laboratory of Proteomics for Drug Discovery, Center for Drug Design Research, National Institutes of Biomedical Innovation, Health and Nutrition, 7-6-8 Saito-Asagi, Ibaraki, Osaka 567-0085, Japan; Laboratory of Proteome Research, National Institutes of Biomedical Innovation, Health and Nutrition, 7-6-8 Saito-Asagi, Ibaraki, Osaka 567-0085, Japan; Laboratory of Proteomics for Drug Discovery, Center for Drug Design Research, National Institutes of Biomedical Innovation, Health and Nutrition, 7-6-8 Saito-Asagi, Ibaraki, Osaka 567-0085, Japan; Laboratory of Proteome Research, National Institutes of Biomedical Innovation, Health and Nutrition, 7-6-8 Saito-Asagi, Ibaraki, Osaka 567-0085, Japan; Laboratory of Proteomics for Drug Discovery, Center for Drug Design Research, National Institutes of Biomedical Innovation, Health and Nutrition, 7-6-8 Saito-Asagi, Ibaraki, Osaka 567-0085, Japan; Laboratory of Proteome Research, National Institutes of Biomedical Innovation, Health and Nutrition, 7-6-8 Saito-Asagi, Ibaraki, Osaka 567-0085, Japan; Graduate School of Informatics, Kyoto University, 36-1 Yoshida-honmachi, Sakyo, Kyoto 606-8501, Japan; Graduate School of Informatics, Kyoto University, 36-1 Yoshida-honmachi, Sakyo, Kyoto 606-8501, Japan; Graduate School of Informatics, Kyoto University, 36-1 Yoshida-honmachi, Sakyo, Kyoto 606-8501, Japan; Ochanomizu University, 2-1-1 Otsuka, Bunkyo-ku, Tokyo 112-8610, Japan; Graduate School of Science and Technology, Nara Institute of Science and Technology (NAIST), 8916–5 Takayama, Ikoma, Nara 630–0192, Japan; Graduate School of Science and Technology, Nara Institute of Science and Technology (NAIST), 8916–5 Takayama, Ikoma, Nara 630–0192, Japan; Graduate School of Science and Technology, Nara Institute of Science and Technology (NAIST), 8916–5 Takayama, Ikoma, Nara 630–0192, Japan; Laboratory of Bioinformatics, Artificial Intelligence Center for Health and Biomedical Research, National Institutes of Biomedical Innovation, Health and Nutrition; 3-17 Senrioka-shinmachi, Settsu, Osaka 567-0085, Japan; Laboratory of Bioinformatics, Artificial Intelligence Center for Health and Biomedical Research, National Institutes of Biomedical Innovation, Health and Nutrition; 3-17 Senrioka-shinmachi, Settsu, Osaka 567-0085, Japan; Laboratory of Bioinformatics, Artificial Intelligence Center for Health and Biomedical Research, National Institutes of Biomedical Innovation, Health and Nutrition; 3-17 Senrioka-shinmachi, Settsu, Osaka 567-0085, Japan; Laboratory of Bioinformatics, Artificial Intelligence Center for Health and Biomedical Research, National Institutes of Biomedical Innovation, Health and Nutrition; 3-17 Senrioka-shinmachi, Settsu, Osaka 567-0085, Japan; Center for Mathematical Modeling and Data Science, the University of Osaka, 1-3 Machikaneyama, Toyonaka, Osaka 560-8531, Japan; Laboratory of Bioinformatics, Artificial Intelligence Center for Health and Biomedical Research, National Institutes of Biomedical Innovation, Health and Nutrition; 3-17 Senrioka-shinmachi, Settsu, Osaka 567-0085, Japan; Department of Electrical Engineering, Waseda University, 3-4-1 Okubo, Shinjuku-ku, Tokyo 169-8555, Japan; Laboratory of Bioinformatics, Artificial Intelligence Center for Health and Biomedical Research, National Institutes of Biomedical Innovation, Health and Nutrition; 3-17 Senrioka-shinmachi, Settsu, Osaka 567-0085, Japan; Institute for Protein Research, the University of Osaka, 3-2 Yamada-oka, Suita, Osaka 565-0871, Japan; Department of Medical Informatics, the University of Osaka Graduate School of Medicine, 2-2 Yamada-oka, Suita, Osaka 565-0871, Japan; Osaka National Hospital, 2-1-14 Hoenzaka, Chuo-ku, Osaka, Osaka 540-0006, Japan; Laboratory of Bioinformatics, Artificial Intelligence Center for Health and Biomedical Research, National Institutes of Biomedical Innovation, Health and Nutrition; 3-17 Senrioka-shinmachi, Settsu, Osaka 567-0085, Japan; Institute for Protein Research, the University of Osaka, 3-2 Yamada-oka, Suita, Osaka 565-0871, Japan; Department of Respiratory Medicine and Clinical Immunology, the University of Osaka Graduate School of Medicine, 2-2 Yamada-oka, Suita, Osaka 565-0871, Japan; Department of Immunopathology, WPI, Immunology Frontier Research Center (iFReC), the University of Osaka, 3-1 Yamada-oka, Suita, Osaka 565-0871, Japan; Integrated Frontier Research for Medical Science Division, Institute for Open and Transdisciplinary Research Initiatives (OTRI), the University of Osaka, 2-1 Yamada-oka, Suita, Osaka 565-0871, Japan; Center for Infectious Disease for Education and Research (CiDER), the University of Osaka, 2-2 Yamada-oka, Suita, Osaka 565-0871, Japan; RIKEN Center for Advanced Intelligence Project, RIKEN, 1-4-1 Nihonbashi, Chuo-ku, Tokyo 103-0027, Japan; NTT Communication Science Laboratories, NTT Corporation, 2-4 Hikaridai, Seika-Cho, Soraku-gun, Kyoto 619-0237, Japan

**Keywords:** IPF, patient stratification, drug target discovery

## Abstract

Idiopathic pulmonary fibrosis (IPF) is an intractable lung disease that belongs to idiopathic interstitial pneumonia (IIP) with limited therapeutic options. Conventional patient stratification approaches often fail to integrate diverse data modalities, particularly heterogeneous electronic medical records (EMR) containing mixed discrete and continuous values, with omics data, or fail to extract the interpretable many-to-many relationships crucial for precision medicine. We introduce subset binding (SB), a novel unsupervised algorithm that extends fuzzy association rule mining to robustly integrate heterogeneous clinical data (EMR) and omics data. This framework is uniquely designed to identify clinically meaningful patient subgroup patterns and discover associated molecular signatures based on observable symptoms rather than relying on ambiguous conventional diagnostic categories, such as IIPs. Applying SB to a dataset including 602 samples (from 403 IIPs including IPF patients and 39 healthy controls), we successfully identified 20 proteins linked with key IPF clinical features. Network-based pathway analysis nominated tyrosine kinases as critical drug target candidates, leading to the proposal of ponatinib, a multi-kinase inhibitor, as a candidate therapeutic. Functional validation using a TGF-β-induced epithelial-mesenchymal transition (EMT) model confirmed ponatinib’s ability to at least partially suppress TGF-β-induced EMT. This inhibitory effect is consistent with the anti-fibrotic mechanism of the existing IPF drug, nintedanib, and reinforces prior evidence supporting ponatinib’s anti-fibrotic property. This study demonstrates that SB enables transparent, reproducible, and robust, molecularly defined patient stratification from multimodal patient data. By establishing a data-driven framework that focuses on observation-based rules, this work lays the critical foundation for future prognostic validation and tailored treatment strategies, offering clinically actionable insights and therapeutic discovery in diagnostically ambiguous diseases like IPF, with ponatinib emerging as a compelling repurposing candidate.

**Significance statement**

Idiopathic pulmonary fibrosis (IPF) is a progressive lung disease with limited therapeutic options. IPF is classified as idiopathic interstitial pneumonia (IIP), but distinguishing it from other similar diseases in IIP is not straightforward. The ambiguities in distinguishing IPF from other IIPs necessitate the identification of molecules associated with specific clinical features, rather than relying on solely on diagnosis. Existing methods for multi-omics data analysis often fail to effectively integrate heterogeneous data – such as EMR (containing mixed discrete and continuous values) and omics – or to extract many-to-many molecular-phenotypic relationships. We developed subset binding (SB), a novel, interpretable unsupervised machine learning method to specifically address these technical limitations by integrating EMR and omics data. Our approach successfully detected proteins in serum extracellular vesicles associated with IPF-related features, highlighted several tyrosine kinases as potential drug targets, and proposed the multi-kinase inhibitor ponatinib as a compelling candidate for drug repurposing. This data-driven framework establishes a scalable and interpretable foundation for biomarker and drug target discovery for intractable diseases whose mechanisms are not fully understood.

## Graphical Abstract

**Graphical Abstract:**
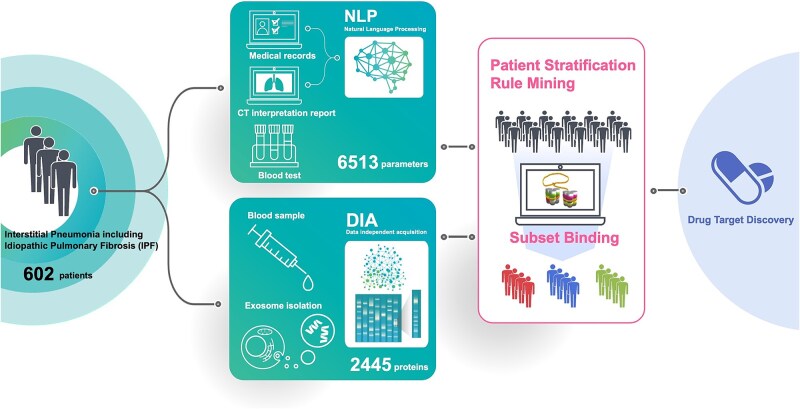

## Introduction

In drug discovery, one of the major challenges is the high failure rate of proof of concept (POC) in Phase II clinical trials, largely owing to the lack of significant efficacy [1]. A key limitation arises from using animal models for drug target discovery, which often fails to translate to humans. Furthermore, patient stratification poses another challenge, as even patients with the same diagnosis can vary significantly in prognosis, treatment response, and risks of side effects [2]. Therefore, stratified therapeutic approaches and drug targets are necessary. By identifying many-to-many relationships between disease phenotypes and biomolecules in a data-driven manner through an integrated analysis of medical information and omics data, we can obtain biomarker candidates. Furthermore, disease phenotypes of patient subgroups can be stratified using these biomolecules. The benefits could be specifically significant for intractable diseases where the molecular mechanisms of disease development are not fully understood. This approach allows for drug target discovery by collecting and analyzing data even when existing knowledge is limited.

Idiopathic pulmonary fibrosis (IPF) is a chronic, progressive, and intractable respiratory disease that belongs to idiopathic interstitial pneumonia (IIP). IIP refers to interstitial pneumonia with no identifiable cause characterized by a usual interstitial pneumonia (UIP) pattern on histopathology. Among its subtypes, IPF has an extremely poor prognosis, and only two treatment options are available, namely the antifibrotic agents pirfenidone and nintedanib [3, 4]. In IIPs, clear diagnostic boundaries are often lacking due to overlapping clinical, radiological, and pathological features, and a multidisciplinary discussion is strongly recommended for a reliable diagnosis. In addition, individual differences have been reported in responses to the aforementioned antifibrotic drugs and the severity of side effects [5]. As a result, supervised learning-based approaches such as classification may have limited applicability, since the ground truth labels themselves may not reflect distinct or biologically coherent disease subtypes. Therefore, the stratification of patients according to the type of IIPs or IPF would be effective in optimizing treatment strategies and obtaining insights into the development of new drugs.

Proteomic profiles of extracellular vesicles (EVs) including exosomes are promising biomarker sources. We have reported several biomarkers for patient stratification or evaluation of disease severity in refractory respiratory diseases such as chronic obstructive pulmonary disease [6] and sarcoidosis [7], which were identified by proteomic analysis of exosomes that contain a complex cargo of contents derived from the original cell, including proteins [8]. The expression of these molecules is closely related to the cell state and the progression of diseases [9, 10]. These discoveries have garnered significant interest in the search for biomarkers using EVs and their applications in drug discovery.

In this study, the core challenge lies in integrating heterogeneous clinical data (which inherently contains mixed data types: discrete values like diagnosis/symptoms and continuous values like laboratory tests) with omics data. We aimed to identify IPF drug target candidates by integrating serum EV proteomics and electronic medical record (EMR) data without utilizing diagnostic label as a prior knowledge. We developed a novel machine learning framework, ‘subset binding (SB)’, to discover interpretable patterns linking omics signatures to patient characteristics for patient stratification and potential drug targets. By combining SB-based rule discovery with network-based drug target discovery, we identified tyrosine kinases as drug target candidates and ponatinib as a potential repurposing candidate. These findings not only offer mechanistic insights into IPF molecular basis but also propose a scalable framework for data-driven precision medicine in complex chronic diseases.

## Materials and methods

### Ethics statement

Written informed consent was obtained from all patients before the study. The study protocol was approved by the ethics committees of the National Institute of Biomedical Innovation (NIBIO) and Osaka University Hospital (Approval number 187). This study adhered to the guidelines issued in the Declaration of Helsinki. Patients diagnosed with or suspected to have interstitial pneumonia, including IPF, at Osaka University Hospital were enrolled in this study. They were provided sufficient explanation based on the ‘Informed Consent Explanation Document.’ The patients then provided written consent to participate in this study.

### Protocol for collecting serum

A total of 10 ml of blood was collected and allowed to stand at room temperature for 1 h and centrifuged at 3000 rpm (1500 *g*) for 10 min. The supernatant was separated as serum. The separated serum was immediately frozen and stored in a freezer at −80 °C. Serum was collected in the same manner for HCs (age-range matched healthy subjects who visited due to some respiratory discomfort but were determined to not have any respiratory disease; had the same laboratory and interview results as the patient group) who were not diagnosed with any organic respiratory disease.

### Protocol for proteome analysis

EV isolation and comprehensive protein measurements were performed as described previously [11]. Briefly, small phosphatidylserine-positive EVs mainly containing exosomes were purified from 200 μL of serum using the MagCapture isolation kit (Fujifilm Wako Pure Chemical Corp.). Our EV isolation and characterizations were performed according to the MISEV2018 guidelines [12]. The average EV sizes and the electron microscope images of the EVs obtained by our method can be found in a previous report [11]. The proteins in the EVs were reduced with tris(2-carboxyethyl) phosphine, alkylated with iodoacetamide, digested with trypsin, and desalted. The pre-treated samples were subjected to LC–MS/MS analysis using the data-independent acquisition (DIA) method. Data analysis was performed using the DIA analysis software Spectranout (Biognosys AG), and runwise imputation was performed for missing value imputation. One commercial serum sample was added to every 15 samples as a quality control to assure quality from sample preparation to data analysis. DIA analysis of the digested HeLa S3 cells (TKG0444, Cell Resource Center for Biomedical Research, Institute of Development, Aging and Cancer, Tohoku University) was performed as a quality control for mass spectrometry. HeLa S3 cells were grown in DMEM containing 10% fetal bovine serum at 37 °C and 5% CO_2_. To visualize the global structure of the proteome data, we used *seaborn* Python module [13], *scikit-learn* Python module [14] and *umap* Python module [15]. Detailed parameters and settings are described in the Supplementary Methods.

### Protocol for collecting medical information

Medical information securely stored in the data center of Osaka University Hospital was anonymized by patient ID. Medical examination records were obtained as structured data from doctors using a template created with a list of 54 items (Supplementary Table 1) of necessary information in advance or by manually structured from free-text data. The CT imaging interpretation reports were tagged with keywords using manual or NLP techniques (Supplementary Methods) and linked with the information about site/lesion pairs and three categories according to the meaning of the sentence: positive (e.g. ‘The presence of honeycombing was observed in the bilateral lower lobes of the lung’ - > ‘honeycombing’ is linked to ‘bilateral lower lobes’ and ‘positive’), negative (e.g. ‘The presence of honeycombing was not observed in the bilateral lower lobes of the lung’ - > ‘honeycombing’ is linked to ‘bilateral lower lobes’ and ‘negative’), and suspect (e.g. ‘The presence of honeycombing was suspected in the bilateral lower lobes of the lung’ - > ‘honeycombing’ is linked to ‘bilateral lower lobes’ and ‘suspect’). Through this procedure, we obtained 6282 items (Supplementary Table 1). The blood test values were structured by selecting and curating 177 key items (Supplementary Table 1). For the initial medical questionnaire and basic information, key items were curated and added to the template items in the medical records. In structuring the data, we confirmed the meaning of the missing values and mainly used reference values for healthy subjects to impute missing values because we determined that the missing data in medical information are often because the data was deemed unnecessary by physicians in the context of diagnosis or other clinical decisions.

### Algorithm of subset binding and data analysis

To find interrelated attributes in heterogeneous data according to their co-occurrence, SB utilized the fuzzy association rule mining approach [16, 17] to search for frequent item sets (items that tend to occur) in two datasets (e.g. proteome data and medical information) and establish association rules (patterns of co-occurrence between item sets), such that the antecedent was derived from one dataset and the consequent from the other (Fig. 3). The detailed algorithm is described in the Supplementary Methods. In the conventional association rule mining approach, association rules are generated ‘within’ each frequent itemset. Our method aims to generate association rules by which their antecedents are derived from one dataset and consequents from the other, so that the detected association rules represent itemsets derived from different datasets related to each other.

Let *I_1_* = {*i_1,1_*, *i_1,2_*, …, *i_1,p_*} of *p* attributes and *I_2_* = {*i_2,1_*, *i_2,2_*, …, *i_2,q_*} of *q* attributes be sets of ‘items’ and *I_1_* and *I_2_* be called ‘itemsets.’ Let *T_1_* = {*t_1,1_*, *t_1,2_*, …, *t_1,m_*} and *T_2_* = {*t_2,1_*, *t_2,2_*, …, *t_2,m_*} be sets of *m* observations in which each of *t_1_* and *t_2_* has *I_1_* and *I_2_*, respectively. *T_1_* and *T_2_* are assumed to have the same number of observations, and *t_1,a_* and *t_2,a_* (*a*∈{1, 2, …, *m*}) are associated with each other (e.g. *t_1,a_*: medical record of patient ID *a*, *t_2,a_*: gene expression profile of patient ID *a*). If *T_1_* and/or *T_2_* contain(s) quantitative attributes, the values were converted into ‘membership values’ for category ‘Low’ and category ‘High’ by applying them to membership functions we designed (‘histogram-based conversion’ and ‘z-score-based conversion’).

First, the fuzzy Apriori algorithm (Apriori algorithm [18, 19] for a fuzzy approach [20],) detects frequent itemsets in *T_1_* and *T_2_* independently with user-specified minimum support. Support can be expressed as follows:

*support* (X → Y) = Σ_(*a*∈{1, 2, …, *m*})_ min(X(*a*), Y(*a*))/*m*

where.

X(*a*): a membership value of X in observation *a*

Y(*a*): a membership value of Y in observation *a*

Second, association rules are generated, so that antecedents are selected from the frequent itemsets detected in *T_1_*, and consequents are selected from those detected in *T_2_*, or vice versa. Several scores can be used as thresholds to limit the number of rules to the output; e.g. lift, confidence and conviction can be expressed as follows:

*lift* (X → Y) = *support* (X → Y)/{*support* (X) * *support* (Y)}

*confidence* (X → Y) = *support* (X → Y) / *support* (X)

*conviction* (X → Y) = (1 – *support* (Y)) / (1 - *confidence* (X → Y))

where.

X: a frequent itemset constituted of *I_1_*

Y: a frequent itemset constituted of *I_2_*

*support*(X) = Σ_(*a*∈{1, 2, …, *m*})_ X(*a*) /*m*

*support*(Y) = Σ_(*a*∈{1, 2, …, *m*})_ Y(*a*) /*m*

The proteome data linked with the medical information were analyzed using SB with the following parameter settings: min support = 0.15, 0.02, 0.02, and 0.02 for the proteome, CT interpretation report, medical records, and blood test, respectively; min number of items = 4, 3, 3, and 3 for the proteome, CT interpretation report, medical records, and blood tests, respectively; min lift = 2, 2, and 2 for the association rules between the proteome and CT interpretation report, proteome and medical records, and proteome and blood tests, respectively. In SB, the strength of association is evaluated using the standard *lift* metric, as commonly employed in association rule mining. A lift value greater than 1 indicates that the observed co-occurrence is stronger than expected by chance, suggesting a non-random association between clinical and molecular features. SB employs rule pruning based on minimum support to mitigate overfitting, following standard practices in association rule mining. We intentionally avoided controlling for demographic and treatment-related confounders prior to analysis, as these variables were considered integral to capturing meaningful stratification patterns. After extracting proteins linked to IPF-associated clinical items using SB, we selected ‘core molecules’ defined as molecules that were linked with all three or two clinical data categories analyzed in this study (Medical Records, CT Interpretation Reports, Blood Test Data).

### Analysis of protein–protein interaction

Protein–protein interaction (PPI) networks for the top 20 proteins were constructed, and network hubs were assigned using TargetMine [21–23]. Hub molecules were defined as the top four proteins with the highest degree centrality within the TargetMine-generated PPI network of the 20 SB-identified proteins.

The network analysis and upstream regulator characterization using IPA (QIAGEN, Redwood City, CA, USA) were used to identify biologically relevant molecular networks and pathways for the core and hub proteins. We performed disease and pathway analyses and network generation using the Ingenuity Knowledge Base. Causal network analysis (CNA) [24] to identify novel master regulators by creating pathways for literature-based relationships.

### Immunohistochemical staining

The fibrotic and normal areas of the lungs of two patients with IPF who had concurrent lung cancer and were eligible for surgery at Osaka University Hospital were separated and fixed in a formalin phosphate buffer solution.

Formalin-fixed lung tissue was cut along the longitudinal axis of the tissue section to prepare formalin-fixed paraffin-embedded blocks. These blocks were thinly sliced to 4 μm in thickness using a sliding microtome to prepare unstained specimens. Unstained specimens were subjected to hematoxylin–eosin (H&E) and Masson’s trichrome (MT) staining to confirm inflammation and fibrosis. In addition, unstained specimens were subjected to immunohistochemistry (IHC) using specific antibodies against the proteins identified in the analysis. The OptiView DAB Universal Kit (Roche Diagnostics, Basel, Switzerland) was used as the detection reagent, and staining using antibody dilutions as a negative control for each antibody was performed simultaneously to evaluate the IHC staining for each antibody. Immunostained tissue specimens were processed using a hybrid cell-counting system equipped with an all-in-one microscope (Keyence).

### Epithelial-mesenchymal transition

EMT was evaluated by suppressing the expression of the epithelial marker E-cadherin and enhancing the expression of the mesenchymal markers, α-SMA, fibronectin and Snail. BEAS-2B cells purchased from ATCC (CRL-9609) at concentrations of 2.5 × 10^4^ or 3.5 × 10^4^ cells/ml were seeded in 96-well plates coated with fibronectin/collagen I/BSA in BEGM™ BulletKit™ (Bronchial epithelial cell basal medium, Lonza Corporation). After 24 h, the test drug was introduced. After 48 h following TGF-β addition, BEADS-2B cells in culture were harvested and adjusted to a concentration of 1 × 10^5^ cells/ml using passaging medium. The cell suspension was aliquoted into tubes to achieve final concentrations of 0.1, 0.18, 0.35, 0.5, 0.7, and 1 × 10^5^ cells/ml. After centrifugation, the supernatant was removed, followed by cell lysis and reverse transcription reactions. Cell lysis and RT were performed using the SuperPrep® II Cell Lysis & RT Kit for qPCR (Toyobo Co., Ltd.), and the expression of the aforementioned EMT marker genes was measured. The expression levels of the markers were normalized using GAPDH expression as a standard. THUNDERBIRD Probe qPCR Mix (Toyobo Co., Ltd.) was used for qPCR, and a TaqMan Probe (Thermo Fisher Scientific Co., Ltd.) was used for each marker probe. Regarding the number of cells used for treatment, no reduction in Cp values was observed at a concentration of 1 × 10^5^ cells/ml compared to 0.7 × 10^5^ cells/ml, suggesting that the PCR had reached saturation. Therefore, the optimal number of cells for treatment was determined to be 0.7 × 10^5^ cells/ ml or lower. This cell count corresponds to a nearly confluent state in a 96-well plate.

## Results

### Data collection from electronic medical record information and structuring with natural language processing

**Figure 1:**
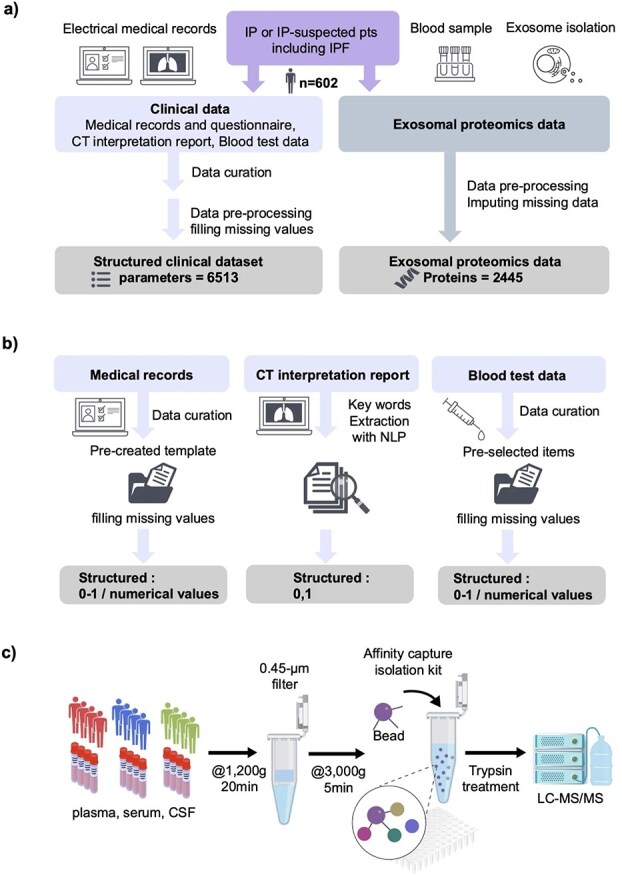
**Workflow for data collection used in this study. a**) Flowchart of medical information and serum sample collection. **b**) Conceptual diagram of medical data generation. **c**) Exosome isolation and proteome analysis.

**Figure 2:**
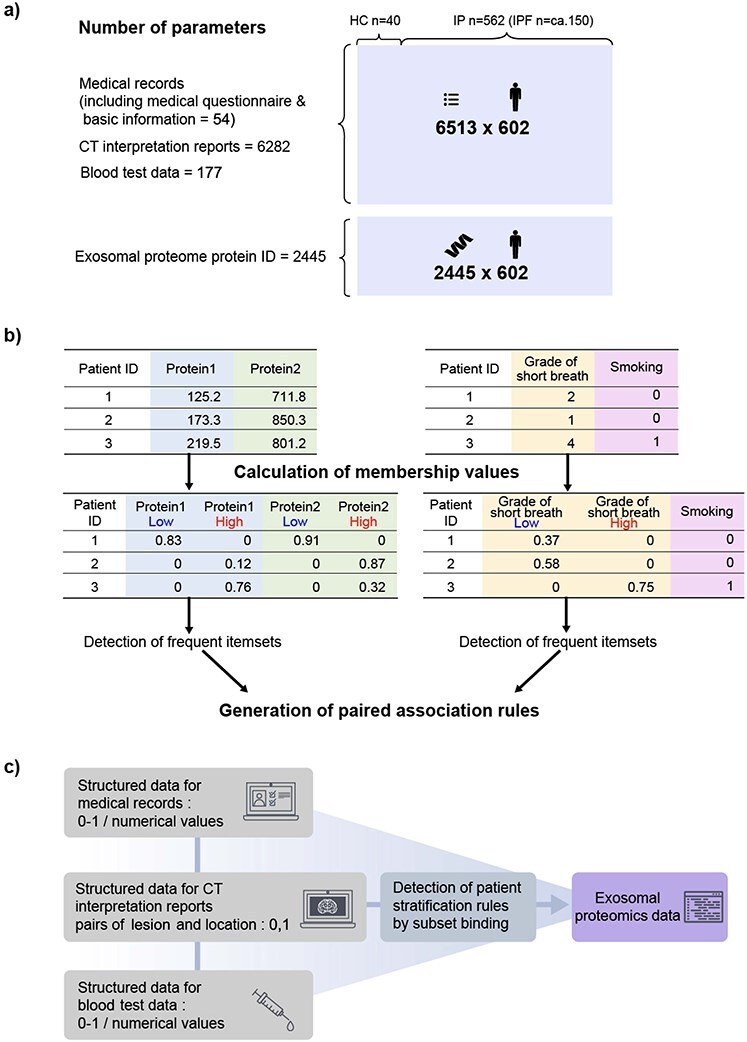
**Data utilized in this study and the corresponding analysis workflow. a**) Dimensions of the structured data. The medical information matrix comprised 6282 attributes from CT interpretation reports, 177 attributes from blood test results, and 54 attributes from medical records. The proteome matrix comprised 2445 proteins (2388 proteins were mapped to known protein IDs as shown in Supplementary Table 2). Both matrices had 602 rows, equivalent to the number of cases (602 cases from 403 patients with IIP and 39 HCs). **b**) Conceptual diagram of SB. Input data: Two paired matrices (quantitative and/or categorical). First, quantitative attributes in the input were converted into fuzzy categorical attributes (‘Low’ and ‘High’) with membership functions. Since membership values for ‘low’ and ‘high’ categories were obtained for each attribute, this process produced matrices with double the number of columns when all attributes were quantitative. Next, these matrices were used to detect frequent item sets independently. Thereafter, association rules were generated such that the frequent item sets derived from one matrix were the antecedent and those from the other matrix were the consequent. The user-specified threshold (e.g. lift) was used for pruning, and paired (antecedent from dataset 1 and consequent from dataset 2) association rules were obtained as output. Examples of output rules are shown in Supplementary Datasets. **c**) Conceptual diagram of the analysis workflow. Since SB accepts two paired matrices, we generated paired association rules by running SB for three specific combinations: i) proteome-medical records, ii) proteome-CT interpretation reports, iii) proteome-blood test. The results from these independent runs (I, ii, iii) were subsequently combined to obtain the IPF-related proteins.

The overall flow of data collection is shown in Fig. 1a,b outlines the procedure from the collection of clinical data through electronic medical record entries (medical records and initial medical questionnaires, CT imaging interpretation reports, and blood test data) to input data generation. The medical records were entered at the time of medical consultation using a format (referred to as a template) created with items set in advance or the information was extracted manually from the entries and initial medical history questionnaire freely written in natural language into the template. The CT imaging interpretation report was paired by natural language processing (NLP) with information about the entity related to the lesion, the site where it was observed and information regarding whether the lesion was observed (positive), not observed (negative), or suspected (suspected) (Supplementary Methods). These features were manually curated and expressed as one-hot vectors for subsequent analyses. Clinical information was collected on or near the date of blood collection for proteomic data acquisition. For proteomic data, the constituent proteins were comprehensively measured via mass spectrometry (Fig. 1c). Finally, we obtained 6513 (6282 attributes from CT image interpretation reports, 177 attributes from blood test results, and 54 attributes from medical records) × 602 cases (with overlap from 403 patients and 39 controls) of medical information and 2445 protein IDs × 602 case matrices (Fig. 2a).

### Basic patient characteristics and clinical items

The basic patient characteristics are presented in Table 1. The collected medical information is listed in Supplementary Table 1, and the 2388 protein groups identified in the proteomic analysis (2445 proteins detected and 2388 proteins mapped to known protein IDs) are shown in Supplementary Table 2.

**Table 1:** Basic patient characteristics.

	Case (Suspected IIP)						Healthy control
	UIP	Probable UIP	Indeterminate UIP	Alternative	Others	Overall	
Number	83	59	38	145	78	403	39
F/M	20/63	12/47	10/28	63/82	55/23	160/243	22/17
Age (average)	70.6	75.0	70.5	68.2	66.2	69.5	67.6

UIP: Usual Interstitial Pneumonia (histopathological finding in IPF).

### Concept of SB and analysis workflow

**Figure 3:**
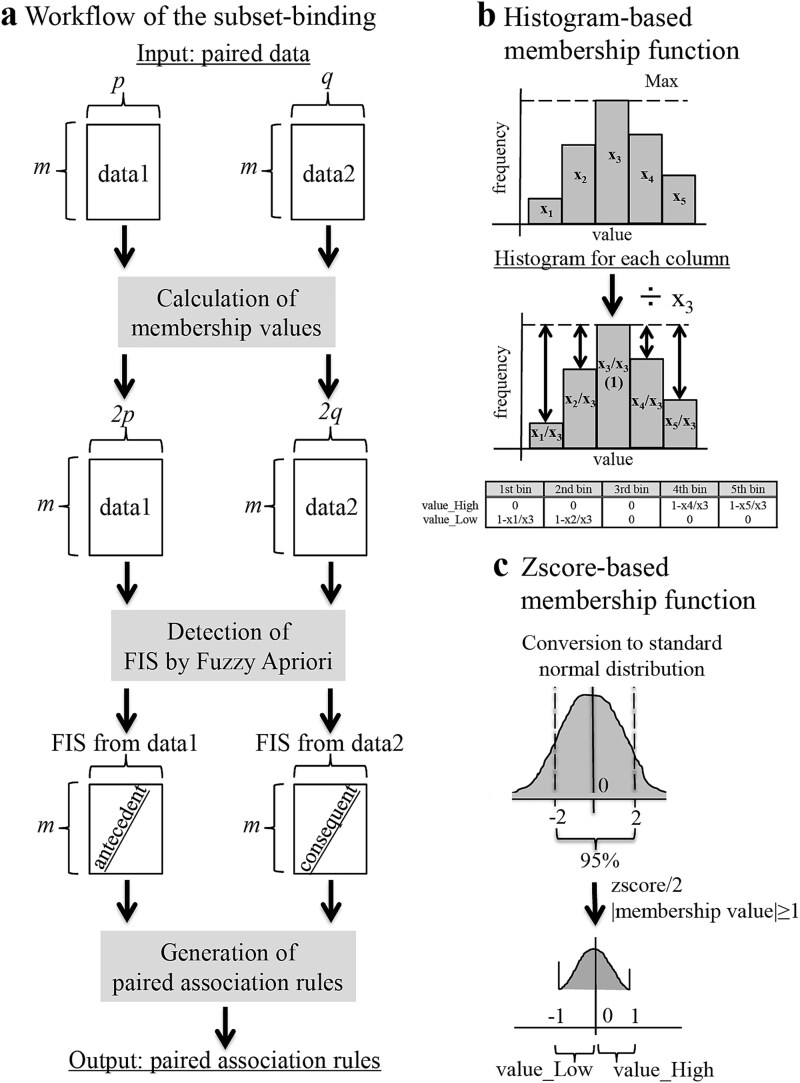
**Workflow of subset binding algorithm and membership functions utilized.**  **a**) Workflow of the subset binding algorithm. Input data: two paired matrices (quantitative and/or categorical). First, quantitative attributes in input are converted into fuzzy categorical attributes (‘Low’ and ‘High’) with membership functions. Since membership values for ‘Low’ and ‘High’ categories are obtained for each attribute, this process produces matrices with double number the of columns when all attributes are quantitative. Next, these matrices are used to detect FIS (frequent itemsets) independently. Thereafter, association rules are generated so that FIS derived from one matrix will be antecedent, and those from the other matrix will be consequent. User-specified threshold (e.g. lift) is used for pruning and paired (antecedent from data1 and consequent from data2) association rules are obtained as output. **b**) Histogram-based membership function. For each attribute (column of input data), a histogram is produced with a user-specified number of bins (e.g. 5). Let the frequency of each bin be x1, x2, x3, x4, and x5, respectively, and x3 be the largest. The formulas to calculate membership values of ‘Low’ and ‘High’ categories are shown at the bottom of Fig. 3b. c) Z-score-based membership function. For each attribute (column of input data), values are converted into z-scores. To scale them between −1 and 1, the z-scores are divided by 2, and values bigger than 1 and those smaller than −1 are converted to 1 or − 1, respectively. The obtained values are used as membership values of ‘Low’ and ‘High’ categories.

The composition of the cohort dataset used for the analysis is shown in Fig. 2a, and the analysis workflow is shown in Fig. 2b. SB, a newly developed unsupervised algorithm, was used to discern patient-stratification rules using structured medical information and proteomic data. SB output patient-stratification rules (e.g. patients with high expression of biomolecules A, B, and C tend to show reticular shadows and traction bronchiectasis) by detecting associations between two input matrices (e.g. proteome data and structured medical information; the number of rows must be the same, but the number of columns may be different) (Figs 2b and 3; see Supplementary Methods for the details of the algorithm). Using this algorithm, data with a mixture of continuous and discrete values, which is common in medical information, can be handled without requiring special preprocessing or prior knowledge. Six combinations of SB analysis are possible, as shown in Fig. 2c. We selected proteins that were included in the IPF characteristic-related association rules in the output of (i) medical records (mixture of binary and numerical values)–protein (numerical values) association, (ii) CT interpretation reports (binary)–protein association, and (iii) blood test (mixture of binary and numerical values)–protein association at least once. The IPF characteristics used to select association rules comprised known biomarkers KL-6 (sialylated carbohydrate antigen) [25] and surfactant protein D (SP-D) in the blood tests; UIP, Probable UIP, honeycombing, traction bronchiectasis and reticular opacity in CT interpretation reports; and dyspnea on exertion, fine crackles and digital clubbing in medical records.

### Clustering of proteome data is not suitable for patient stratification

**Figure 4:**
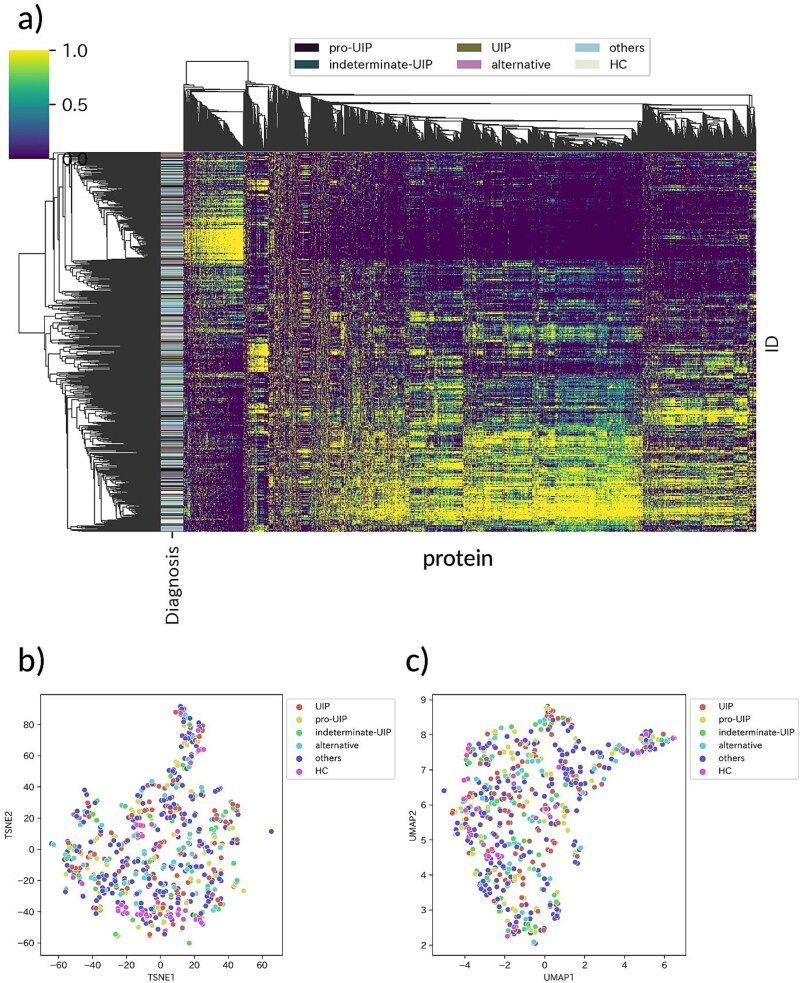
**Visualization of the proteome data. a**) Heat map with hierarchical clustering. The log-transformed protein amounts are scaled for each column. The X-axis represents proteins detected by DIA (n = 2445), and the Y-axis represents the cases (n = 602). The diagnosis of the cases has been represented in six different colors, as shown at the top of the figure. UIP: Usual interstitial pneumonia (histopathological finding in IPF), pro-UIP: Probable UIP, HC: Health control. **b**) t-SNE. The log-transformed and scaled protein amounts are plotted (metric: Cosine, perplexity: 5). **c**) UMAP. The log-transformed and scaled protein amounts are plotted (metric: Cosine, perplexity: 5). These results indicate that clustering based solely on proteomic profiles is insufficient for meaningful patient stratification. Furthermore, the lack of clear separation among interstitial pneumonia subtypes suggests that diagnosis-based supervised approaches may have limited utility in capturing underlying biological heterogeneity.

To investigate whether global similarities in the proteomic data of serum EVs reflected the diagnosis, we visualized their quantitative patterns using a heatmap with hierarchical clustering (Fig. 4a), t-SNE (Fig. 4b), and UMAP (Fig. 4c). The heatmap shows that the global similarities among cases did not match their diagnoses, which implies that canonical approaches, such as clustering, are not suitable for patient stratification. This indicated that the proteome data contained many proteins that were not directly linked to phenotypes, such as diagnosis. Fig. 4b and c also support this tendency, in which several subtypes in IIPs (UIP, probable UIP, indeterminate UIP, alternative, and others) did not show colocalization. In contrast, healthy controls (HCs) showed a weak tendency to colocalize.

Canonical machine learning techniques assume high global similarities among cases when diagnosis or phenotypic characteristics are alike. Notably, SB was able to uncover latent data structures that remained undetected by bi-clustering methods in synthetic benchmark datasets (Supplementary Methods). Therefore, we concluded that clustering is not suited to extract disease phenotype-related proteins. We performed SB to identify proteins linked with IPF characteristics (Fig. 2b), which resulted in the identification of 20 proteins.

### Exploring the relationship between findings of IPF-associated characteristics and serum exosomal proteins

**Figure 5:**
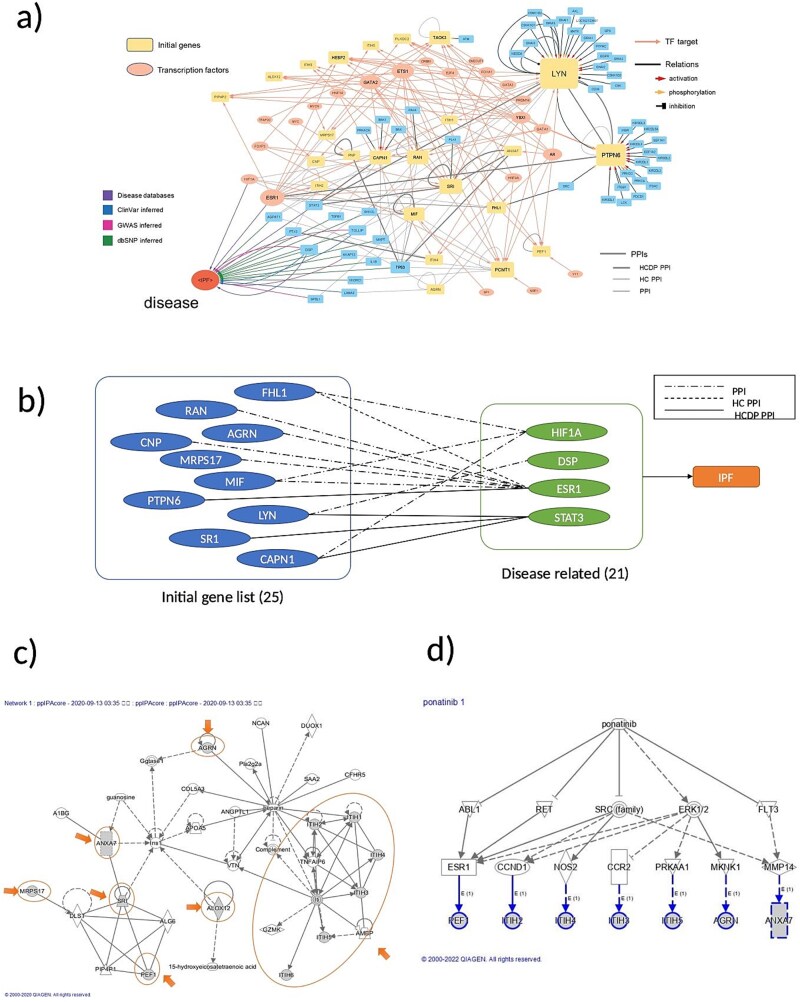
**Network-based analysis of IPF-related proteins identified by SB.** A functional interaction network constructed using proteins associated with IPF-related clinical items detected via SB. Nodes represent proteins and edges denote known functional associations. **a**) Functional network of SB-identified proteins and hub proteins obtained by TargetMine. The color and shape of nodes correspond to molecular categories (e.g. transcription factors, SB-derived proteins referred as initial genes). See figure key for details. High-confidence (HC) PPI; PPIs that were supported by at least two different experimental methods or two independent publications. High-confidence direct physical (HCDP) PPI; PPIs were defined as a subset of the high-confidence PPIs that were annotated as physical interaction by data sources, or the supported by experiments, such as yeast two-hybrid assays, that can detect direct physical interactions. **b**) Subnetwork linking IPF and SB-identified proteins. This figure shows a subnetwork extracted from the full network shown in Fig. 5a, consisting of the IPF node, proteins directly connected to IPF, and the proteins identified by SB that are connected to those IPF-associated proteins. The edge style represents the confidence level of the protein–protein interactions (PPI, HC PPI, HCDP PPI). **c**) Regulatory pathway network of core molecules by IPA. The regulatory network that contains the core molecules was obtained by IPA analysis. The core molecules identified by SB are positioned in close proximity within the regulatory network. The network implies potential importance in the clinical features that are observed in IPF. Node shape: molecular classes. **d**) Upstream regulatory cascade of SB-identified proteins, targetable tyrosine kinases and ponatinib. This cascade diagram illustrates the upstream regulators of the core proteins identified by SB. The SB-identified core molecules are shown to be regulated by several common tyrosine kinases, indicating that these kinases may serve as potential therapeutic targets. Furthermore, existing knowledge reveals that these tyrosine kinases are targets of ponatinib. In c) and d), Node shape: molecular classes. Node color: predicted activation state inferred by IPA (orange: activated, blue: inhibited). Solid lines represent direct molecular interactions, whereas dashed lines indicate indirect relationships. Arrowheads denote activation, and blunt-ended lines denote inhibition. SB-identified core molecules are highlighted with bold outlines.

The 20 IPF-related proteins identified by SB are listed in Table 2. Protein–protein interrelationships among the 20 molecules were searched using TargetMine [21–23]. The tyrosine-protein kinase LYN, tyrosine-protein phosphatase non-receptor type 6 (PTPN6), macrophage migration inhibitory factor (MIF), and GTP-binding nuclear protein Ran (RAN) were found to be the hub molecules in the protein–protein interactions among these 20 molecules (Fig. 5a). In addition, the presence or absence of a relationship between the 20 molecules was explored using TargetMine and ingenuity pathway analysis (IPA, QIAGEN) (Fig. 5b and Supplementary Table 3). Molecules with no previously reported association were 28S ribosomal protein S17, mitochondrial (MRPS17) and peflin (PEF1), whereas molecules that were associated with IPF through many other molecules were LYN, PTPN6, MIF, and RAN.

**Table 2:** List of proteins co-occurring with medical information reflecting IPF characteristics.

Protein name	Gene symbol	Correlated with		
		Medical records	CT interpretation reports	Blood test data
Annexin A7	ANXA7	◯	◯	◯
Inter-alpha-trypsin inhibitor heavy chain	ITIH4	◯	◯	◯
28S ribosomal protein S17, mitochondrial	MRPS17	◯	◯	◯
Agrin	AGRN	◯	◯	◯
Sorcin	SRI	◯	◯	
Polyunsaturated fatty acid lipoxygenase	ALOX12	◯	◯	
Peflin	PEF1	◯	◯	
Purine nucleoside phosphorylase	PNP	◯		
Four and a half LIM domains protein1	FHL1	◯		
Protein-L-isoaspartate(D-aspartate) O-methyltransferase	PCMT1	◯		
Type 2 phosphatidilinositol 4,5-bisphosphate 4-phosphatase	PIP4P2	◯		
Heme-binding protein 2	HEBP2	◯		
Calpain-1 catalytic subunit	CAPN1	◯		
Plexin domain-containing protein 2	PLXDC2	◯		
Tyrosine-protein phosphatase non-receptor type 6	PTPN6			◯
Tyrosine-protein kinase Lyn	LYN			◯
Serine/threonine-protein kinase TAO3	TAOK3			◯
GTP-binding nuclear protein Ran	RAN			◯
2′,3′-cyclic-nucleotide 3′-phosphodiesterase	CNP			◯
Macrophage migration inhibitory factor	MIF			◯

For benchmarking, we conducted a comparative analysis using MOFA2 [26] and Similarity Network Fusion (SNF), established unsupervised algorithms for multi-omics data integration, to identify feature pairs across the two different datasets. In the MOFA2 analysis, while some associations were successfully identified—notably including the proteins ITIH4 and ANXA7, which were also detected by SB—a significant limitation was observed. 57 proteins were selected as the MOFA2 results (Supplementary Table 4, Supplementary Methods). The numbers of pairs between proteome data and other datasets were 0 for CT interpretation report, 47 for medical record, and 298 for blood test. The numbers of unique features were four for medical record, and 21 for blood test, and all of these features were continuous values. MOFA2 predominantly combined features with continuous values but failed to effectively integrate binary features with continuous ones. Furthermore, SNF failed to identify any distinct clusters in this dataset (Supplementary Methods).

### Network analysis and search for upstream regulatory factors

In addition, we identified molecular networks composed of the seven core molecules. We found pathways, such as carbohydrate metabolism, small-molecule biochemistry, and cellular assembly and organization, where all seven molecules were mapped (Fig. 5c). The core molecules are regulated by proteins such as estrogen receptor 1 (ESR1), cyclin D1 (CCND1), C-C chemokine receptor type 2 (CCR2), nitric oxide synthase 2 (NOS2), and matrix metalloproteinase-14 (MMP14), which are, in turn, regulated by the proto-oncogene tyrosine-protein kinase Src (SRC) family, extracellular signal-regulated kinase 1/2 (ERK1/2), and ABL proto-oncogene 1 (ABL1) (Fig. 5d). Finally, ponatinib was identified as an upstream master regulator. The MGI (http://www.informatics.jax.org/) and JAXKO mouse phenotype databases (https://www.jax.org/jax-mice-and-services) were used to search for KO mice and the phenotypes of core and hub molecules, which are summarized in Supplementary Table 5. LYN and PTPN6 were associated with phenotypes such as lung inflammation.

### Immunohistochemical staining revealed a remarkable upregulation in the expression of several proteins in fibrotic areas, especially in epithelial cells and inflammatory cells, in an independent cohort

**Figure 6:**
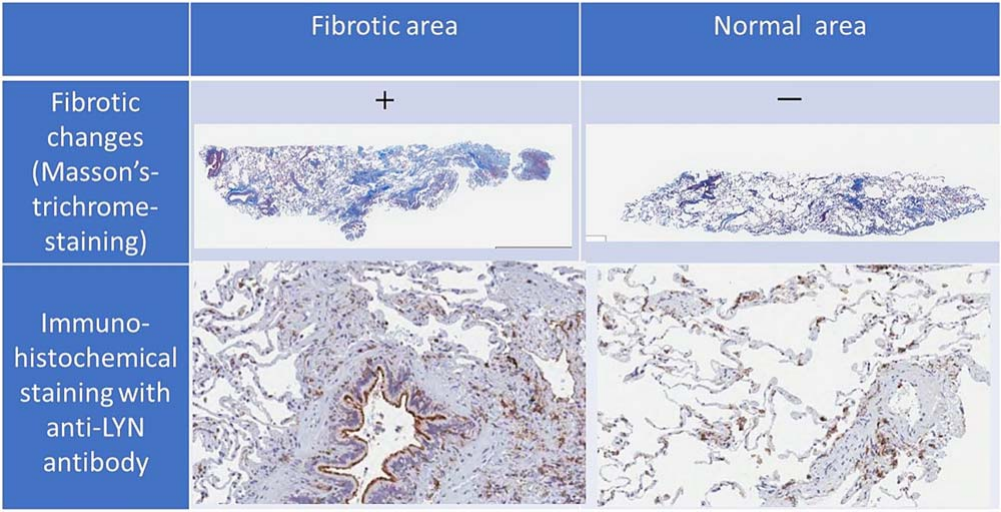
**Immunohistochemical validation of IPF-related protein expression in IPF fibrotic lesions (independent cohort).** Representative immunohistochemical images for proteins identified via SB. Distinct spatial expression patterns in IPF lung sections support the proteomics signatures discovered computationally. The core protein identified by SB, LYN, shows a tendency toward increased expression in fibrotic regions of the lung. The summary of the statistics of the results of immunohistochemical staining for all the core molecules are shown in Supplementary Table 6.

To support the biological validity of the SB-derived signatures, we performed independent validation using immunohistochemistry, demonstrating spatial expression in fibrotic lesions. The seven core proteins and four hub proteins were investigated for their expression in the patient’s lungs and their increased expression in fibrotic areas. The fibrotic and normal areas of the lungs of two newly recruited patients with IPF who had concomitant cancer and were eligible for surgery were used for immunostaining with antibodies against each protein. The samples were sourced from a separate cohort distinct from that used for proteome analysis. As a representative image, a clear enhancement of LYN expression was observed in tissues with obvious fibrosis, as confirmed via Masson’s trichrome staining (Fig. 6). All proteins except ALOX12 were upregulated in fibrotic areas, especially in epithelial and inflammatory cells (Supplementary Table 6).

### Ponatinib suppressed epithelial–mesenchymal transition

**Figure 7:**
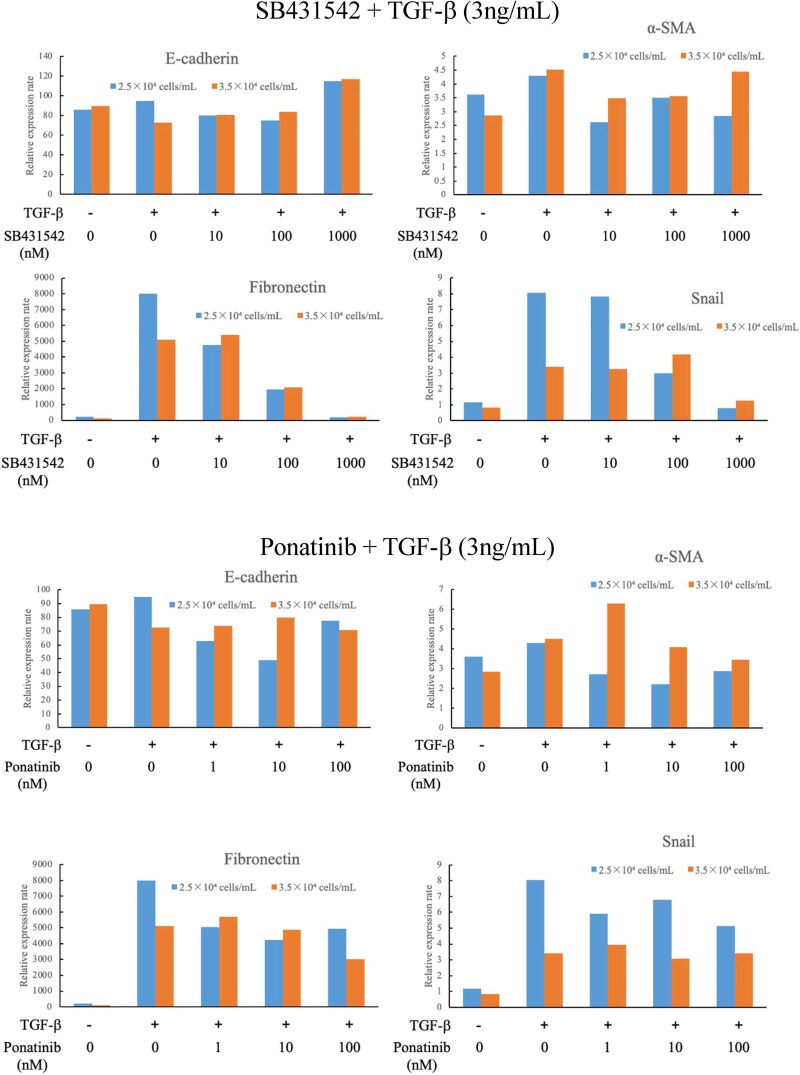
**Ponatinib treatment attenuated TGF-β-induced expression of EMT genes/markers.** Cells were treated with ponatinib or SB431542. Subsequently, rTGF-β was added to the cells and cultured for 48 h. Furthermore, cells were subjected to RT-qPCR for the specified transcripts of **a**) E-cadherin, **b**) α-SMA, **c**) fibronectin, and **d**) Snail. During the progression of EMT, the expression of E-cadherin and α-SMA showed little change in our EMT model, while the expression of fibronectin and Snail was upregulated. Treatment with the positive control SB431542 (upper panel) attenuated the upregulation of fibronectin and Snail in a dose-dependent manner. A similar dose-dependent pattern was observed upon treatment with ponatinib (lower panel), suggesting that ponatinib inhibits TGF-β-induced expression of EMT at least partially, aligning with the network analysis. Relative expression levels are presented as the means.

To further support the biological validity of the SB-derived signatures, we performed epithelial-mesenchymal transition (EMT) assay, demonstrating the functional impact on fibrosis-related pathways. EMT is an important mechanism underlying pulmonary fibrosis in IPF. BEAS-2B human normal airway epithelial cells were treated with ponatinib or SB431542 (selective inhibitor of TGF-β type I receptor kinase, used as positive control), after which rTGF-β was added to the cells to induce EMT, and the transcripts of the established markers for EMT, E-cadherin, α-SMA, fibronectin and Snail, were determined (Fig. 7). When EMT is induced by TGF-β, the expression of fibronectin and Snail was elevated, while the expression of E-cadherin and α-SMA showed little change. Upon the treatment of SB431542, EMT was suppressed as expected, leading to a reduction in the expression of fibronectin and Snail. Similarly, the treatment of ponatinib exhibited trends comparable to those observed with SB431542, the positive control. These findings suggest that ponatinib possesses inhibitory effects on EMT at least partially.

## Discussion

In this study, we focused on proteins in serum EVs owing to their low invasiveness and potential to reflect the pathology of respiratory diseases. As biomolecules in exosomes are transferred between cells [27], they can help elucidate the pathogenesis underlying diseases, including malignant diseases [9], immune diseases [28], and infectious diseases [29], as well as physiological conditions [8]. We confirmed that the expression levels of proteins linked to the major phenotypic characteristics of IPF were high in fibrotic lung areas (Fig. 6). This highlights the relevance of these proteins in disease pathogenesis as well as validates the use of our data-driven drug target discovery approach. By focusing on EV-encapsulated proteins, we minimized the impact of other proteins that are abundantly found in the blood and identified proteins that were closely related to disease pathology.

Even when structured medical information and corresponding omics data are collected, conventional machine learning methods may not always be effective for specific objectives. Medical data often contains a complex mixture of discrete values (e.g. binary indicators for smoking, disease progression stages, and items representing the findings observed in CT images as one-hot vectors) and continuous values (e.g. blood test values and respiratory function measurements). Existing methods, such as MOFA [30], MOFA2 [26] and DIABLO [31], while these methods are powerful for extracting ‘global’ latent factors shared across datasets, they may overlook ‘local’ associations restricted to specific patient subgroups. In addition, SB offers a distinct advantage in handling heterogeneous data types to discover latent patterns that conventional integration methods miss. We emphasize that SB is uniquely positioned as a rule-mining approach designed to detect these local, many-to-many relationships (e.g. specific symptoms linking to specific protein subsets) that are critical for stratifying heterogeneous diseases like IIP including IPF, which global clustering methods failed to capture (Fig. 4). We performed PCA, t-SNE, and UMAP using the IPF-associated proteins identified by MOFA2 (Supplementary Fig. 1) and SB (Supplementary Fig. 2). However, consistent with the global analysis in Fig. 4a, the IIP subtypes and HCs remained indistinguishable without clear separation in both cases. The fact that these proteins—which are biologically validated as drug target candidates (e.g. linked to Ponatinib/EMT inhibition)—do not form global clusters reinforces our argument: standard stratification methods fail to capture these clinically relevant but locally restricted signals, necessitating rule-based approaches like SB.

Since SB is an unsupervised method that does not rely on predefined class labels, conventional performance metrics such as F1-score are not applicable. Instead, we validated the algorithmic output using biological experiments and clinical interpretability. We detected proteins known to be associated with IPF as well as those not yet linked to the disease (Supplementary Table 3). The fact that our method detected the ground truth supports its validity.

Notably, the proteins identified in this study were linked to a common pathway, with ponatinib emerging as a drug regulating this pathway. Using a TGF-β-induced EMT system, we found that ponatinib inhibited EMT at least partially (Fig. 7). The concept of partial EMT, where epithelial cells acquire mesenchymal features without full transition, has recently emerged as relevant in tissue fibrosis [32]. This partial EMT was observed in silica-induced lung fibrosis and knockdown of Snail inhibited EMT [33]. Since ponatinib inhibited the upregulation of Snail together with fibronectin by TGF- β and this effect was comparable to the positive control SB431542, our result strongly indicates that ponatinib has an anti-fibrotic property. This inhibitory effect on EMT is highly consistent with the findings obtained with nintedanib, an anti-fibrotic drug used in IPF treatment, which similarly inhibits EMT [34]. Nintedanib is a tyrosine kinase inhibitor targeting vascular endothelial growth factor, platelet-derived growth factor, and fibroblast growth factor. Ponatinib is also a multi-targeted tyrosine kinase inhibitor [35]. Importantly, ponatinib has previously been shown to inhibit the apoptosis of human type I alveolar epithelial cells and the proliferation of human lung fibroblasts *in vitro*, and to suppress bleomycin-induced pulmonary fibrosis in rats via the TGF-β1/Smad3 pathway [36]. Furthermore, ponatinib has been shown to suppress EMT in A549 cells [36]. These combined external evidences strongly support our SB-driven hypothesis that ponatinib is a viable repurposing candidate for IPF.

## Conclusion

Taken together, our study demonstrates a scalable and interpretable workflow for clinical biomarker and drug target discovery using real-world medical and molecular data. Such findings pave the way for subgroup-specific treatment strategies and identify new therapeutic targets in disease subtypes not adequately addressed by current therapies. While this study presents a robust, critical proof-of-concept framework for identification of clinically meaningful patient subgroup patterns and associated molecular signatures, establishing a powerful new method for heterogeneous data integration, we recognize several key avenues for future methodological application and enhancement. First, the identified subgroups and therapeutic hypotheses generated by SB require subsequent prospective application to prognostic analysis. Future research should leverage the robust, molecularly defined subgroups identified here to rigorously evaluate the prognostic implications or treatment response profiles of identified subgroups, thereby fulfilling prerequisites for optimizing treatment strategies and achieving clinical translation. Second, applying the identified rules and protein signatures to independent multi-center cohorts will be essential to demonstrate the framework’s broad utility and generalizability across diverse clinical settings. A multi-center collaborative study involving IIPs, including IPF, is currently being planned, and data collection is underway. Third, the potential therapeutic effect of ponatinib in IPF, which was partially evaluated *in vitro,* warrants further comprehensive investigation. Future work should focus on *in vivo* studies and subsequent clinical investigations to fully establish its efficacy and safety in IPF. Nonetheless, the study provides the critical molecular and computational groundwork necessary for integrating real-world multimodal data into clinically actionable decision-support frameworks in complex diseases.

**Key Points**

- The subset binding (SB) algorithm is a novel unsupervised method extending fuzzy association rule mining, specifically designed to overcome the challenge of integrating highly heterogeneous clinical data (a mix of discrete and continuous values) with omics data.
- Conventional multi-omics methods are typically unsuitable for robustly extracting the many-to-many associations necessary for patient stratification when dealing with mixed clinical data types.
- Applying the SB framework to interstitial pneumonia including idiopathic pulmonary fibrosis (IPF) successfully identified 20 key serum extracellular vesicle proteins linked to clinical features, enabling drug target discovery via molecularly defined patient stratification based on observable symptoms.
- Network-based analysis, leveraging SB-derived signatures, nominated several tyrosine kinases as critical drug targets and proposed ponatinib, a multi-kinase inhibitor, as a promising drug repurposing candidate for IPF.
- This study establishes a scalable and transparent computational workflow that provides reproducible and clinically actionable molecular insights for therapeutic discovery in diagnostically ambiguous diseases.

## Supplementary Material

Supplementary_material_bbag153

## Data Availability

The proteome data obtained in this study were deposited in the jPOST database (https://globe.jpostdb.org/) with the accession number PXID042707.

## Code availability

The source code of the SB algorithm is not publicly available at this time due to a patent. However, all necessary details of the algorithm and parameter settings are described in the methods and Supplementary Information to ensure conceptual reproducibility.

## Notes

Preprint servers: https://doi.org/10.21203/rs.3.rs-405195/v4 (Research Square, CC BY 4.0 License)

Classification: Physical Sciences (Biophysics and Computational Biology), Biological Sciences (Medical Sciences)
