# Supplementary material for: Subset binding enables detection of multimodal patient subgroup patterns and drug target discovery in idiopathic pulmonary fibrosis
Source: Brief Bioinform. 2026 Apr 14;27(2):bbag153. doi: 10.1093/bib/bbag153 (PMC13076932; doi:10.1093/bib/bbag153)
Supplement: Supplementary_material_bbag153 [file supplementary_material_bbag153.zip › SupplementaryTable3_revise.pdf]

Supplementary Table 3

	Protein name	Gene name	Connection to IPF	#molecules connected	Molecules connected
1	Annexin A7	ANXA7	I(Indirect)	9	PTEN, SRC, VIM, etc.
2	Inter-alpha-trypsin inhibitor heavy chain 4	ITIH4	I	1	ADAM10
3	28S ribosomal protein S17, mitochondrial	MRPS17	N	0	
4	Agrin	AGRN	I	6	TGFb1, SRC, VIM, etc.
5	Sorcin	SRI	I	4	TGFb1, USHBP1, STAT3, etc.
6	Polyunsaturated fatty acid lipoxygenase	ALOX12	I	5	TGFb1, PPARG, PLA2G4A, etc.
7	Peflin	PEF1	N	0	
8	Purine nucleoside phosphorylase	PNP	I	2	TGFb1, miR-30c-5p
9	Four and a half LIM domains protein1	FHL1	I	3	TGFb1, SRC, CTNNB1
10	Protein-L-isoaspartate(D-aspartate) O-methyltransferase	PCMT1	I	3	AGTR1, VHL, PPARG
11	Type 2 phosphatidilinositol 4,5-bisphosphate 4-phosphatase	PIP4P2	I	1	TNF
12	Heme-binding protein 2	HEBP2	I	1	SHMT2
13	Calpain-1 catalytic subunit	CAPN1	I	11	TGFb1, TNF, PTEN, etc.
14	Plexin domain-containing protein 2	PLXDC2	I	2	IGF1, TNF
15	Tyrosine-protein phosphatase non-receptor type 6	PTPN6	I	31	TGFb1, CD44, ABL1,etc.
16	Tyrosine-protein kinase Lyn	LYN	D	31	nintedanib, SRC, FLT3,etc.
17	Serine/threonine-protein kinase TAO3	TAOK3	I	1	miR17-5p
18	GTP-binding nuclear protein Ran	RAN	I	15	TNF, TERT, VHL, ABL1,etc.
19	2',3'-cyclic-nucleotide 3'-phosphodiesterase	CNP	I	3	IGF1, TNF, NPPB
20	Macrophage migration inhibitory factor	MIF	I	19	TGFb1, bleomycin, VHL, etc.
