# Supplementary material for: Subset binding enables detection of multimodal patient subgroup patterns and drug target discovery in idiopathic pulmonary fibrosis
Source: Brief Bioinform. 2026 Apr 14;27(2):bbag153. doi: 10.1093/bib/bbag153 (PMC13076932; doi:10.1093/bib/bbag153)
Supplement: Supplementary_material_bbag153 [file supplementary_material_bbag153.zip › SupplementaryTable4_revise.pdf]

Supplementary Table 4.　IPF-associated protein identified by MOFA2

ID	diff	ProteinNames	description
Q14624-2;Q14624-3	1.62	ITIH4_HUMAN	;
P01700	1.55	LV147_HUMAN	extracellular region,plasma membrane,extracellular exosome,blood microparticle
P02741	1.52	CRP_HUMAN	extracellular region,extracellular space,extracellular exosome
P02652	1.52	APOA2_HUMAN	extracellular region,early endosome,endoplasmic reticulum lumen,cytosol,very-low-density lipoprotein particle,high-density lipoprotein particle,spherical high-density lipoprotein particle,chylomicron,extracellular exosome,blood microparticle
P02647	1.49	APOA1_HUMAN	extracellular region,extracellular space,nucleus,early endosome,endoplasmic reticulum lumen,cytosol,plasma membrane,cell surface,endocytic vesicle,cytoplasmic vesicle,very-low-density lipoprotein particle,high-density lipoprotein particle,discoidal high-density lipoprotein particle,spherical high-density lipoprotein particle,secretory granule lumen,chylomicron,extracellular exosome,endocytic vesicle lumen,blood microparticle,extracellular vesicle
P01009	1.48	A1AT_HUMAN	Golgi membrane,extracellular region,proteinaceous extracellular matrix,extracellular space,endoplasmic reticulum,endoplasmic reticulum lumen,Golgi apparatus,ER to Golgi transport vesicle,platelet alpha granule lumen,endoplasmic reticulum-Golgi intermediate compartment membrane,extracellular exosome
P0DOX2	1.41	IGA2_HUMAN	
P00751	1.41	CFAB_HUMAN	extracellular region,extracellular space,plasma membrane,extracellular exosome,blood microparticle
Q9GZX5	1.41	ZN350_HUMAN	nucleus,nucleoplasm,nuclear matrix,transcriptional repressor complex
P02747	1.40	C1QC_HUMAN	extracellular region,collagen trimer,extracellular space,extracellular exosome,blood microparticle
P01024	1.39	CO3_HUMAN	extracellular region,extracellular space,plasma membrane,extracellular exosome,blood microparticle
Q9Y4I1;Q9Y4I1-2;Q9Y4I1-3	1.39	MYO5A_HUMAN	ruffle,photoreceptor outer segment,cytoplasm,Golgi apparatus,cytosol,intermediate filament,membrane,myosin complex,growth cone,filopodium tip,insulin-responsive compartment,microtubule plus-end,melanosome,actomyosin,neuron projection,neuronal cell body,extracellular exosome,lysosome,early endosome,late endosome,peroxisome,endoplasmic reticulum,actin filament,vesicle,recycling endosome;;
P27918	1.39	PROP_HUMAN	extracellular region,extracellular space,endoplasmic reticulum lumen,extracellular matrix
P02790	1.38	HEMO_HUMAN	extracellular region,extracellular space,extracellular exosome,endocytic vesicle lumen,blood microparticle
Q5T5C0;Q5T5C0-2	1.38	STXB5_HUMAN	cytoplasm,plasma membrane,acetylcholine-gated channel complex,synaptic vesicle,cell junction,secretory granule,cytoplasmic vesicle membrane,SNARE complex;
P01834	1.36	IGKC_HUMAN	extracellular region,extracellular space,plasma membrane,external side of plasma membrane,immunoglobulin complex, circulating,extracellular exosome,blood microparticle
P01023	1.30	A2MG_HUMAN	extracellular region,cytosol,platelet alpha granule lumen,extracellular exosome,blood microparticle
P01876	1.29	IGHA1_HUMAN	extracellular region,extracellular space,external side of plasma membrane,extracellular exosome,monomeric IgA immunoglobulin complex,secretory IgA immunoglobulin complex,secretory dimeric IgA immunoglobulin complex,blood microparticle
P02655	1.28	APOC2_HUMAN	extracellular region,extracellular space,early endosome,very-low-density lipoprotein particle,low-density lipoprotein particle,intermediate-density lipoprotein particle,spherical high-density lipoprotein particle,chylomicron,extracellular exosome
P02765	1.27	FETUA_HUMAN	extracellular region,extracellular space,extracellular matrix,platelet alpha granule lumen,extracellular exosome,blood microparticle
P02787	1.27	TRFE_HUMAN	extracellular region,extracellular space,early endosome,late endosome,clathrin-coated pit,basal plasma membrane,cell surface,endosome membrane,cytoplasmic, membrane-bounded vesicle,apical plasma membrane,endocytic vesicle,extrinsic component of external side of plasma membrane,vesicle,secretory granule lumen,basal part of cell,perinuclear region of cytoplasm,recycling endosome,extracellular exosome,blood microparticle,HFE-transferrin receptor complex
P69891	1.25	HBG1_HUMAN	cytosol,hemoglobin complex
P69905	1.23	HBA_HUMAN	extracellular region,cytosol,hemoglobin complex,membrane,cytosolic small ribosomal subunit,haptoglobin-hemoglobin complex,extracellular exosome,endocytic vesicle lumen,blood microparticle
P13645;SWISS-PROT:P13645	1.22	K1C10_HUMAN;	extracellular space,nucleus,cytoplasm,intermediate filament,membrane,extracellular exosome;
P60709;P63261;SWISS-PROT:P60712	1.19	ACTB_HUMAN;ACTG_HUMAN;	nuclear chromatin,extracellular space,nucleoplasm,cytoplasm,cytosol,cytoskeleton,plasma membrane,focal adhesion,membrane,intracellular ribonucleoprotein complex,cortical cytoskeleton,NuA4 histone acetyltransferase complex,cytoplasmic ribonucleoprotein granule,myelin sheath,protein complex,extracellular exosome,MLL5-L complex,blood microparticle,dense body;extracellular space,nucleus,cytosol,cytoskeleton,plasma membrane,focal adhesion,membrane,myofibril,extracellular matrix,filamentous actin,myelin sheath,extracellular exosome,blood microparticle,dense body;
P08514	1.18	ITA2B_HUMAN	plasma membrane,integral component of plasma membrane,focal adhesion,integrin complex,external side of plasma membrane,cell surface,platelet alpha granule membrane,extracellular exosome,blood microparticle
Q562R1	1.16	ACTBL_HUMAN	extracellular space,cytoplasm,cytoskeleton,extracellular exosome
P62736	1.16	ACTA_HUMAN	extracellular space,cytoplasm,cytosol,actin cytoskeleton,lamellipodium,filopodium,smooth muscle contractile fiber,protein complex,cell body,extracellular exosome

P02743	1.16	SAMP_HUMAN	extracellular region,extracellular space,nucleus,extracellular exosome,blood microparticle,extracellular matrix
P07477;SWISS-PROT:P07477	1.12	TRY1_HUMAN;	extracellular region,extracellular exosome,blood microparticle;
Q66K66	1.08	TM198_HUMAN	plasma membrane,integral component of membrane,cytoplasmic, membrane-bounded vesicle
P0DOX5	1.06	IGG1_HUMAN	
P68871	1.01	HBB_HUMAN	extracellular region,cytosol,hemoglobin complex,haptoglobin-hemoglobin complex,extracellular exosome,endocytic vesicle lumen,blood microparticle
P09871	1.01	C1S_HUMAN	extracellular region,extracellular exosome,blood microparticle
SWISS-PROT:P00761	1.00	TRYP_PIG	
O00712;O00712-4;O00712-5;O00712-6	0.99	NFIB_HUMAN	nucleus,nucleolus,cerebellar mossy fiber;;;
P11142	0.99	HSP7C_HUMAN	Prp19 complex,extracellular space,intracellular,nucleus,nucleoplasm,spliceosomal complex,nucleolus,lysosomal membrane,late endosome,cytosol,plasma membrane,cell-cell adherens junction,focal adhesion,membrane,intracellular ribonucleoprotein complex,extracellular matrix,melanosome,lysosomal lumen,myelin sheath,clathrin-sculpted gamma-aminobutyric acid transport vesicle membrane,extracellular exosome,blood microparticle,lumenal side of lysosomal membrane,presynapse,ubiquitin ligase complex
P00736	0.97	C1R_HUMAN	extracellular region,extracellular exosome,blood microparticle
P02656	0.96	APOC3_HUMAN	extracellular region,extracellular space,early endosome,very-low-density lipoprotein particle,intermediate-density lipoprotein particle,spherical high-density lipoprotein particle,chylomicron,extracellular exosome
P0DOY2	0.95	IGLC2_HUMAN	
P02042	0.82	HBD_HUMAN	cytosol,hemoglobin complex,blood microparticle
P30626;P30626-2;P30626-3	0.74	SORCN_HUMAN	nucleoplasm,cytoplasm,mitochondrion,endoplasmic reticulum membrane,smooth endoplasmic reticulum,cytosol,membrane,sarcoplasmic reticulum,Z disc,T-tubule,sarcoplasmic reticulum membrane,axon terminus,dendritic spine neck,extracellular exosome,chromaffin granule membrane;;
P01871;P01871-2	0.73	IGHM_HUMAN	extracellular space,plasma membrane,external side of plasma membrane,cell surface,integral component of membrane,extracellular exosome,pentameric IgM immunoglobulin complex,hexameric IgM immunoglobulin complex,blood microparticle;
P20073;P20073-2	0.72	ANXA7_HUMAN	nucleus,nuclear envelope,endoplasmic reticulum membrane,cytosol,plasma membrane,membrane,extracellular exosome,chromaffin granule membrane;
O14672	0.72	ADA10_HUMAN	nucleus,cytoplasm,Golgi apparatus,Golgi-associated vesicle,plasma membrane,focal adhesion,cell surface,postsynaptic density,membrane,integral component of membrane,intracellular membrane-bounded organelle,extracellular exosome,perinuclear endoplasmic reticulum,tetraspanin-enriched microdomain
P21926	0.67	CD9_HUMAN	extracellular space,plasma membrane,integral component of plasma membrane,focal adhesion,external side of plasma membrane,membrane,apical plasma membrane,endocytic vesicle membrane,clathrin-coated endocytic vesicle membrane,platelet alpha granule membrane,extracellular exosome,extracellular vesicle
P50995;P50995-2	0.64	ANX11_HUMAN	nuclear envelope,nucleoplasm,cytoplasm,spindle,membrane,midbody,melanosome,specific granule,azurophil granule,phagocytic vesicle,extracellular exosome;
P10909;P10909-2;P10909-4;P10909-5	-0.56	CLUS_HUMAN	extracellular region,extracellular space,nucleus,cytoplasm,mitochondrion,endoplasmic reticulum,Golgi apparatus,cytosol,cell surface,extracellular matrix,platelet alpha granule lumen,mitochondrial membrane,spherical high-density lipoprotein particle,chromaffin granule,protein complex,perinuclear region of cytoplasm,extracellular exosome,blood microparticle,neurofibrillary tangle,apical dendrite;;;
P0DOX6	-0.67	IGM_HUMAN	
P01591	-0.79	IGJ_HUMAN	extracellular region,extracellular space,extracellular exosome,monomeric IgA immunoglobulin complex,dimeric IgA immunoglobulin complex,secretory IgA immunoglobulin complex,secretory dimeric IgA immunoglobulin complex,pentameric IgM immunoglobulin complex,blood microparticle,hexameric IgM immunoglobulin complex
P02654	-0.80	APOC1_HUMAN	endoplasmic reticulum,very-low-density lipoprotein particle,high-density lipoprotein particle,chylomicron,extracellular exosome
P02730	-0.80	B3AT_HUMAN	plasma membrane,integral component of plasma membrane,integral component of membrane,basolateral plasma membrane,Z disc,cortical cytoskeleton,extracellular exosome,blood microparticle
P0DOX8	-0.83	IGL1_HUMAN	
P01859	-0.84	IGHG2_HUMAN	extracellular region,extracellular space,external side of plasma membrane,immunoglobulin complex, circulating,extracellular exosome,blood microparticle
P11166	-0.85	GTR1_HUMAN	Golgi membrane,female pronucleus,cytosol,plasma membrane,integral component of plasma membrane,caveola,cell-cell junction,membrane,basolateral plasma membrane,apical plasma membrane,midbody,cortical actin cytoskeleton,melanosome,extracellular exosome,blood microparticle
P00915	-1.06	CAH1_HUMAN	cytosol,extracellular exosome
P02786	-1.15	TFR1_HUMAN	extracellular region,extracellular space,endosome,plasma membrane,integral component of plasma membrane,clathrin-coated pit,external side of plasma membrane,cell surface,membrane,cytoplasmic, membrane-bounded vesicle,basolateral plasma membrane,melanosome,intracellular membrane-bounded organelle,perinuclear region of cytoplasm,recycling endosome,extracellular exosome,blood microparticle,extracellular vesicle,HFE-transferrin receptor complex
