# Supplementary material for: Subset binding enables detection of multimodal patient subgroup patterns and drug target discovery in idiopathic pulmonary fibrosis
Source: Brief Bioinform. 2026 Apr 14;27(2):bbag153. doi: 10.1093/bib/bbag153 (PMC13076932; doi:10.1093/bib/bbag153)
Supplement: Supplementary_material_bbag153 [file supplementary_material_bbag153.zip › SupplementaryTable5_revise.pdf]

Supplementary Table 5

Phenotype of KO mice for proteins found in subset binding

| Gene Symbol | Abnormal phenotype findings |
|---|---|
| Annexin A7 | (pancreas inflammation) |
| MRPS17 | No data |
| Agrin | Primary atelectasis |
| SRI | (Metabolism, glucose) |
| ALOX12 | Normal |
| Peflin | (Neurological) |
| ITIH4 | Normal |
| LYN | Lung inflammation |
| MIF | Abnormal morphology |
| RAN | Increased carcinoma incidence |
| PTPN6 | IP, inflammation, distress |
