# Supplementary material for: Subset binding enables detection of multimodal patient subgroup patterns and drug target discovery in idiopathic pulmonary fibrosis
Source: Brief Bioinform. 2026 Apr 14;27(2):bbag153. doi: 10.1093/bib/bbag153 (PMC13076932; doi:10.1093/bib/bbag153)
Supplement: Supplementary_material_bbag153 [file supplementary_material_bbag153.zip › SupplementaryTable6_revise.pdf]

Supplementary Table 6

Summary of the results of immunohistochemical staining

Molecular Symbol	Fibrotic Tissue % (N=5)					Average	No Affected Tissue % (N=5)					Average
Annexin A7	9	10	12	6	2	7.8 ±3.04**	2	1	4	2	0	1.8 ±1.04
MRPS17	7	33	13	10	4	13.4 ±7.84*	2	5	1	0	0	1.6 ±1.52
Agrin	6	13	9	15	14	11.4 ±3.12**	1	2	2	0	1	1.2 ±0.64
SRI	8	5	10	7	7	7.4 ±1.28**	2	2	2	0	0	1.2 ±0.96
ALOX12	12	4	13	5	4	7.6 ±3.92	1	5	1	1	0	1.6 ±1.36
Peflin	11	8	7	3	5	6.8 ±2.24**	2	3	1	1	0	1.4 ±0.88
ITIH4	15	10	11	1	4	8.2 ±4.56*	1	4	1	0	0	1.2 ±1.12
LYN	11	8	9	4	5	7.4 ±2.32**	1	1	0	0	0	0.4 ±0.48
MIF	6	6	7	4	4	5.4 ±1.12**	4	1	1	0	1	1.4 ±1.04
RAN	8	12	15	6	14	11.0 ±3.2**	1	4	1	1	0	0.6 ±1.04
PTPN6	10	10	14	8	2	8.8 ±3.04*	1	1	0	1	0	1.2 ±0.48

*Significance $p<0.05$

**Significance $p<0.01$

Molecular Symbol	Fibrotic Tissue[1]	No Affected Tissue[1]
Annexin A7	7.8 ±3.04**	1.8 ±1.04
MRPS17	13.4 ±7.84*	1.6 ±1.52
Agrin	11.4 ±3.12**	1.2 ±0.64
SRI	7.4 ±1.28**	1.2 ±0.96
ALOX12	7.6 ±3.92	1.6 ±1.36
Peflin	6.8 ±2.24**	1.4 ±0.88
ITIH4	8.2 ±4.56*	1.2 ±1.12
LYN	7.4 ±2.32**	0.4 ±0.48
MIF	5.4 ±1.12**	1.4 ±1.04
RAN	11.0 ±3.2**	0.6 ±1.04
PTPN6	8.8 ±3.04*	1.2 ±0.48

[1]Average of five (%)

*Significance $p<0.05$

**Significance $p<0.01$
