# Supplementary material for: Subset binding enables detection of multimodal patient subgroup patterns and drug target discovery in idiopathic pulmonary fibrosis
Source: Brief Bioinform. 2026 Apr 14;27(2):bbag153. doi: 10.1093/bib/bbag153 (PMC13076932; doi:10.1093/bib/bbag153)
Supplement: Supplementary_material_bbag153 [file supplementary_material_bbag153.zip › SupplementaryFigures.pdf]

a)

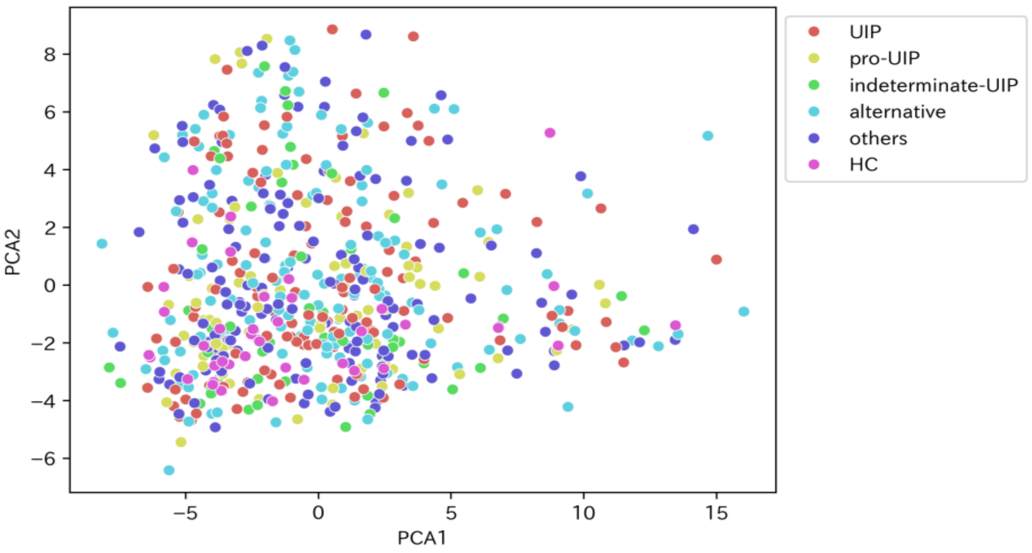

b)

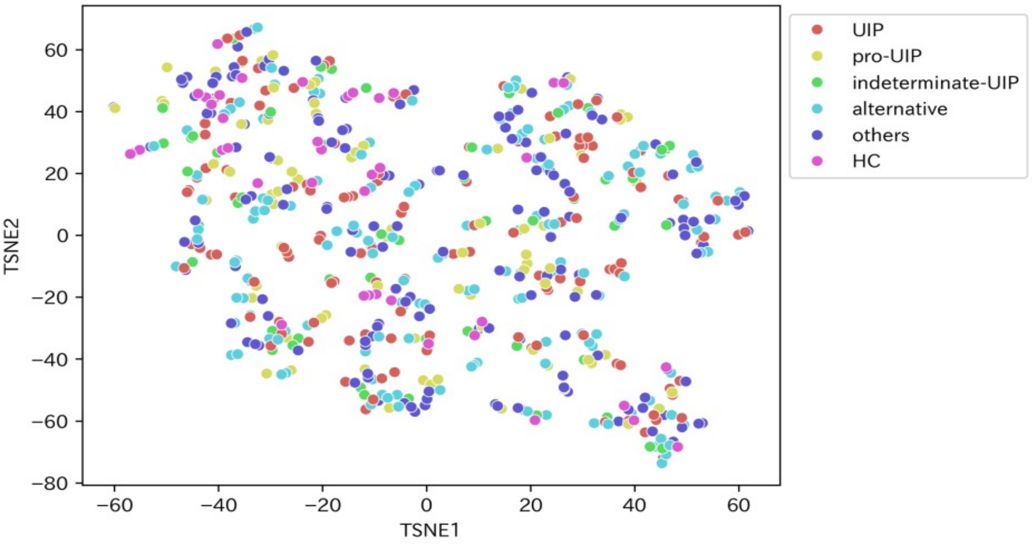

c)

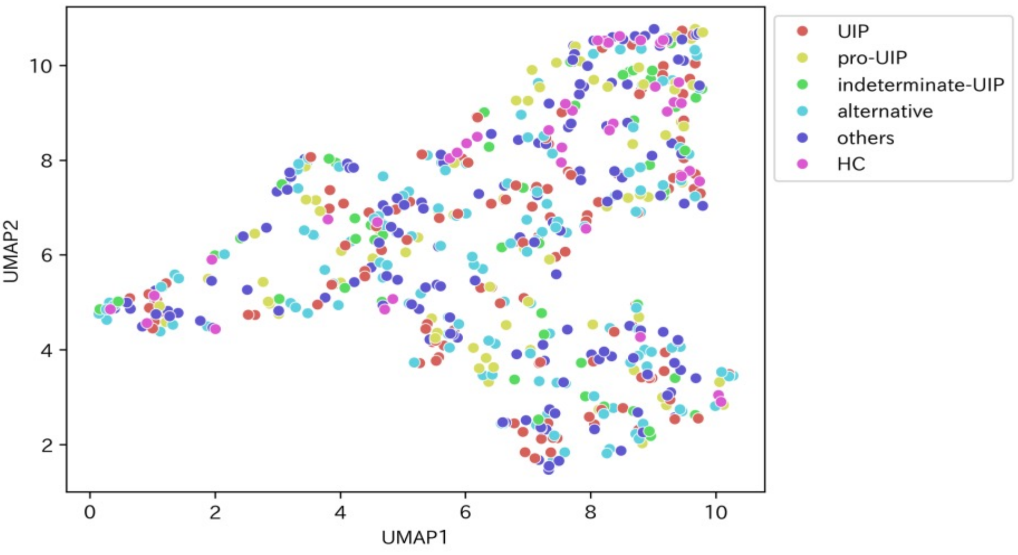

Supplementary Figure 1 Visualization of the proteome data (57 proteins detected by MOFA2). a) PCA. **b)** t-SNE. The log-transformed and scaled protein amounts are plotted (metric: cosine, perplexity: 5). **c)** UMAP. The log-transformed and scaled protein amounts are plotted (metric: cosine, perplexity: 5). These results indicate that the profiles of 57 IPF proteins detected by MOFA2 is still insufficient for meaningful patient stratification.

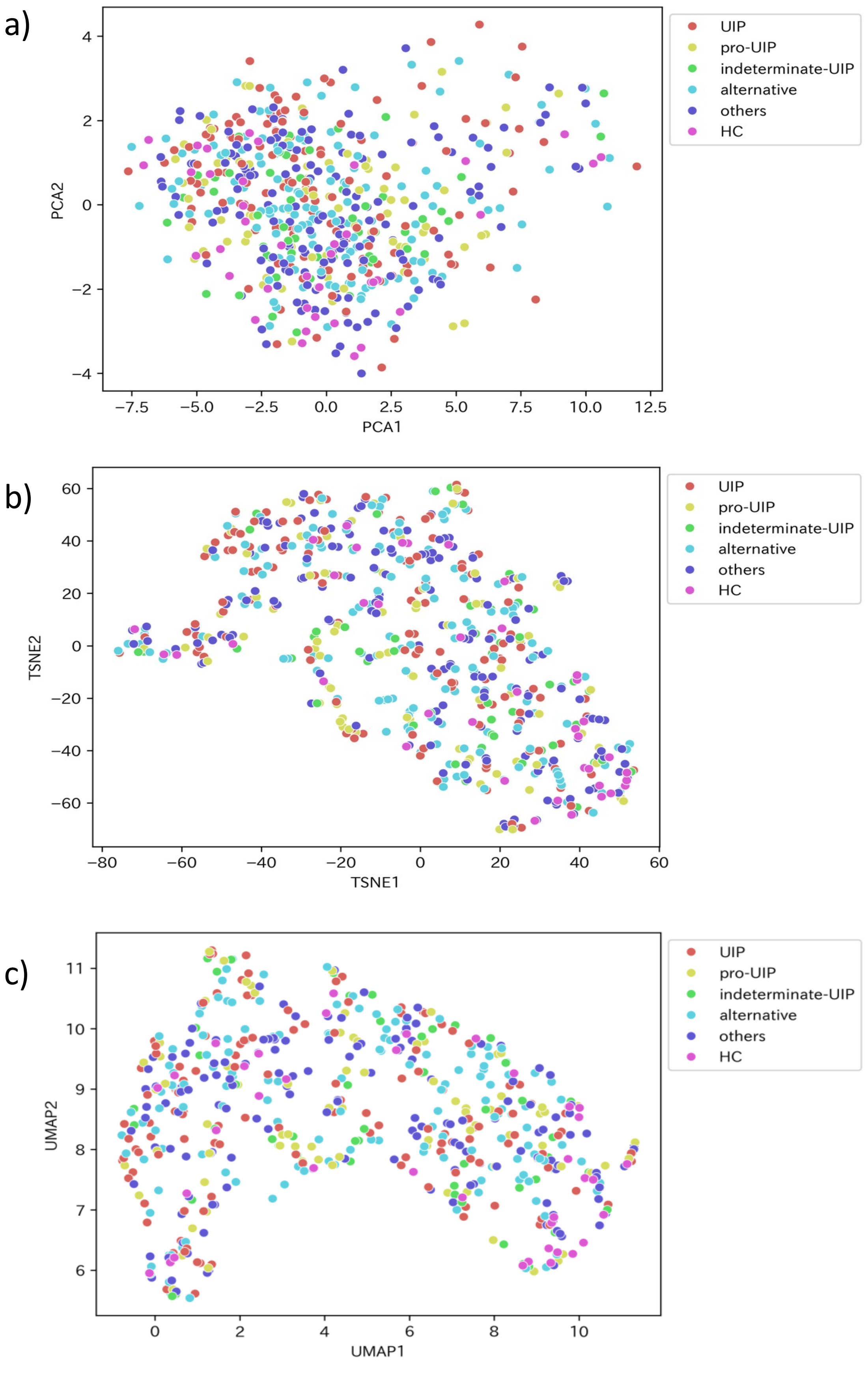

Supplementary Figure 2 Visualization of the proteome data (20 IPF-related proteins). a) PCA. **b)** t-SNE. The log-transformed and scaled protein amounts are plotted (metric: cosine, perplexity: 5). **c)** UMAP. The log-transformed and scaled protein amounts are plotted (metric: cosine, perplexity: 5). These results indicate that the profiles of 20 IPF-related proteins is still insufficient for meaningful patient stratification.
