# Supplementary material for: Subset binding enables detection of multimodal patient subgroup patterns and drug target discovery in idiopathic pulmonary fibrosis
Source: Brief Bioinform. 2026 Apr 14;27(2):bbag153. doi: 10.1093/bib/bbag153 (PMC13076932; doi:10.1093/bib/bbag153)
Supplement: Supplementary_material_bbag153 [file supplementary_material_bbag153.zip › SupplementaryMethods_revise.docx]

**Supplementary Methods**

**Subset Binding algorithm: Description and Implementation**

**Algorithm in details**

Our study aimed to develop a method that would enable i) the search of frequent itemsets in a given dataset containing continuous values and ii) finding association rules between frequent itemsets that are detected in different given datasets to associate two heterogeneous datasets focusing on a limited number of items, by extension of fuzzy association rule mining (FARM).

$I=\left\{ i_{1}, i_{2},\ldots, i_{n} \right\}$ $T=\left\{ T_{1}, T_{2},\ldots, T_{m} \right\}$ $X,Y\subseteq I$ $X\cap Y=\emptyset$ $support\left( X\to Y \right)=\left( \left| X\cup Y \right| \right)/m.$Conventional association rule mining (ARM) approaches assume that input data contain categorical attributes. However, the data that we handle can be quantitative or a mixture of qualitative and quantitative data. Therefore, quantitative attributes are converted into categorical attributes by putting thresholds for quantization (e.g., when threshold1 and threshold2 are given, and threshold1 < threshold2, quantitative attributes can be assigned to one of these categories: i) ≤ threshold1, ii) between threshold1 and threshold2, and iii) ≥ threshold2).

This forms the crisp data; however, the procedure results in loss of information. To solve this problem, fuzzy logic is introduced in the Apriori algorithm.

Fuzzy logic is defined as “a class of objects with a continuum of grades of membership,” and quantitative attributes are converted into several categories with “membership values” ranging from 0 to 1 (e.g., quantitative attribute at threshold1 is converted into i) 0.5 category and ii) 0.5 category). The notions of union, intersection, and complement, which are used to calculate several important scores in ARM, such as *support*, can also be extended to fuzzy sets.

**Membership functions for calculation of membership values**

When converting quantitative attributes into fuzzy categorical sets, the main problem lies in defining membership functions, which are used to calculate membership values. Because membership values range from 0 to 1, *min-max* scaling, sigmoid transformation, or rank-based conversion is typically used. However, these methods reduce the differences in membership values between the most applicable category and the least applicable category and obtain too-fuzzy data for the Apriori algorithm. With this preliminary observation, we designed novel membership functions, as described next.

**・Histogram-based conversion**

The frequency of quantitative data is converted into a histogram with a user-specified number of bins. The quantitative attributes are converted into three categories: “low,” “average,” and “high.” Their membership values for quantitative attribute *v* can be expressed as given subsequently, where the frequency of the bin that includes the quantitative attribute *v* is F*_v_*, frequency of the highest bin is F_H_, lower boundary of the highest bin is b_L_, and upper boundary of the highest bin is b_H_.

Membership value for category “low” = 1 - F*_v_*/F_H_ if *v* < b_L_, (4)

1. otherwise; (5)

Membership value for category “high” = 1 – F*_v_*/F_H_ if *v* > b_H_, (6)

0 otherwise. (7)

The sum of membership values for categories “low,” “average,” and “high” is supposed to be 1. However, the information in category “average” will not be used for ARM because it will be handled as frequently occurring items and will fail to detect interesting association rules that include category “low” or category “high.”

**・Z-score-based conversion**

The frequency of quantitative data was converted into a standard normal distribution to obtain z-scores, the quantitative attributes are converted into membership values in three categories: “low,” “average,” or “high” with the membership functions, which divide z-scores by 2 because approximately 95% of the data is known to range from -2 to 2 and make them range from -1 to 1 by adding Wisorization. Their membership values for quantitative attribute *v* can be expressed as follows:

Membership value for category “low” (*v*) = -(z-score(*v*)/2) if -1 ≤ z-score (*v*)/2 < 0 (8)

1 if -(z-score(*v*)/2) > 1 (9)

0 otherwise (10)

Membership value for category “high” (*v*) = z-score(*v*)/2 if 0 < z-score (*v*)/2 ≤ 1 (11)

1 if z-score(*v*)/2 > 1 (12)

0 otherwise (13)

By analogy with the histogram-based conversion, the sum of membership values for categories “low,” “average,” and “high” is supposed to be 1. However, the information in category “average” will not be used for ARM because it will be handled as frequently occurring items and will fail to detect interesting association rules that include category “low” or category “high.”

- **Other membership functions used for comparison**

The formula of min-max scaling for the quantitative attribute *v* is as follows:

Membership value for category “Low” = 1 - (*v* - *v*_min_) / (*v*_max_ - *v*_min_), (14)

Membership value for category “High” = (*v* - *v*_min_) / (*v*_max_ - *v*_min_). (15)

The formula of sigmoid function for the quantitative attribute *v* is as follows:

Membership value for category “Low” = 1 – 1/(1 + e*^-v^*), (16)

Membership value for category “High” = 1/(1 + e*^-v^*). (17)

The formula of rank-based conversion for the quantitative attribute *v* is as follows:

Membership value for category “Low” = 1 – r(*v*)/n, (18)

Membership value for category “High” = r(*v*)/n, (19)

where r is the rank, and n is the number of observations.

**Experiments with artificial and real data**

We performed experiments with two artificial datasets and one real biological dataset to confirm the performance of our algorithm in detecting paired frequent itemsets for subset-binding. Our algorithm can also be applied to arbitrary paired datasets. For simplicity, this study assumed that the dataset consists of gene expression profile data (data1) and clinical measurement data (data2).

- **Artificial data (small)**

Artificial data were generated, as shown in Supplementary figure SM1a. The gene expression profile data had 100 rows (for 100 patients) and 200 columns (for 200 genes), and random values were generated according to a standard normal distribution. Clinical measurement data were generated using the same procedure. We added some irregular patterns to these two matrices, randomly generated according to the normal distribution, with different mean and standard deviation (SD) values, as frequent itemsets to be detected. We then evaluated the performance of the algorithm by confirming whether the irregular patterns we added were successfully detected. The patterns to detect are summarized in Table SM1. With this dataset, we compared five membership functions: min-max scaling, conversion with sigmoid function, rank-based conversion, histogram-based conversion, and z-score-based conversion (details have been provided in the Methods section); Table SM2 summarizes the results. With the parameter settings used, only the histogram-based function and z-score-based function generated paired frequent itemsets, and all the three patterns that we added were included in the generated rules. The other three methods (min-max scaling and sigmoid and rank-based conversions) detected none of them with the parameters tested. We attempted to apply bi-clustering (plaid model) to this dataset. However, this method failed to detect any structure or consistent signals, likely due to the sparse and non-global patterns embedded in the dataset.

- **Artificial data (large)**

Next, we performed an experiment with a large dataset. Paired matrices with 1000 rows and 2000 columns were generated using the same procedure as in the case of artificial data (small), and we added three patterns to be detected, as shown in Figure SM1b and Table SM1. The histogram-based function and z-score-based function successfully detected all the three patterns that we generated in the outputs (Table SM2).

- **Real data (Liver toxicity)**

We validated the usefulness of SB by performing an experiment on a real biological dataset [SM1]. Acetaminophen (50, 150, 1500, and 2000 mg/kg body-weight), which is known to cause liver toxicity at high doses, was administered during a light period to 64 rats (4 male rats per dose group), and they were sacrificed after 6, 18, 24, or 48 h. Hepatic gene expression profiling on the left liver lobe was performed using Agilent-011868 (G4130) rat oligonucleotide microarrays (Agilent Technologies, Palo Alto, CA), and 3116 genes were selected as being significantly differentially expressed in the acetaminophen-treated group by comparing with a control group (Figure SM2a, data1). DAVID [SM10] was used to convert Agilent probe IDs to Entrez gene IDs and gene names. In addition, 48 histopathological observations (qualitative data) and 10 clinical measurements (quantitative data) were obtained from these rats (Figure SM2a, data2). In the original biological data that we used for the experiment (liver toxicity data), histopathological observations were described as “minimal,” “mild,” “moderate,” or “marked.” For this experiment, they were converted into “0,” “1,” “2,” or “3,” respectively. Authors of the original paper had reported 50 and 150 mg/kg body-weight ratios of acetaminophen as subtoxic, and 1500 and 2000 mg/kg body-weight ratios as severely toxic. Hence, the column of dose levels in data2 was converted into binary attributes to represent their toxicity level (50 and 150 mg/kg body-weight: 0; 1500 and 2000 mg/kg body-weight: 1). In addition, the column of time points in data2 was converted into binary attributes to represent their toxicity level (6, 18, 48 h: 0; 24 h: 1) because 24 h was reported to be the time of peak of toxicity, and rats were in a recovery phase after 48 h of acetaminophen treatment.

With the parameter settings that we tested (min support for data1 and data2: 0.02, min items for data1 and data2: 10, lift: 4.8), 3986 paired-association rules were generated. Since many association rules with similar *lift* values were generated, we used *conviction* values in conjunction with lift values for the purpose of ranking in this analysis. The one with the highest conviction is shown in Figure SM2b. The results demonstrated that high values of alkaline phosphatase (ALP), alanine aminotransferase (ALT), aspartate aminotransferase (AST), and total bile acid (TBA) with a low value of cholesterol co-occurred with histopathological observations, as can be seen under the “histopathological observation” column in data2 of Figure SM2b (“LLL_Centrilob_Necrosis,” “LLL_Hepato_Hypertrophy,” “LML_Centrilob_Necrosis,” and “LML_Sinusoid_Congestion”), and were further related to the toxic dose and time point of acetaminophen treatment. These attributes were then paired with 10 probes (“A_43_P10003” (gene name: Hsph1), “A_42_P717602” (gene name: Mat2a), “A_42_P484423” (gene name: Pgs1), “A_43_P17455” (gene name: Dnah9), “A_43_P14864” (gene name: Dynll1), “A_43_P16523” (gene name: Nomo1), “A_43_P19279” (gene name: Lyzl4), “A_43_P12811” (gene name: Srm), “A_42_P655825” (gene name: Smg9), and “A_42_P804499” (n.d.)) in its antecedent. The result was consistent with the fact that high values of ALP, ALT, AST, and TBA and low levels of cholesterol are regarded as markers of liver toxicity, and a high dose of acetaminophen causes liver toxicity.

We confirmed that these outputs were consistent with the previous reports. An overdose of acetaminophen has been reported to cause sinusoidal congestion and centrilobular necrosis [SM2] owing to an intermediate metabolite and could be fatal. In addition, acetaminophen has been reported to cause hepatocyte hypertrophy in rats [SM3]. Furthermore, some of the genes detected in the antecedent were indicated to be involved in liver injury. Methionine adenosyltransferase (Mat) is responsible for the biosynthesis of S-adenosylmethionine (AdoMet) and exists in two isoforms in mammals, namely Mat1a and Mat2a. Mat2a is induced in response to a liver injury, which accelerates cell division and hepatocyte growth [SM4]. AdoMet functions as a precursor of antioxidative glutathione (GSH) and polyamines, which are involved in cell growth and apoptosis. GSH depletion by acetaminophen treatment and its influence on hepatic necrosis have also been studied extensively [SM5]. In addition, spermidine synthase (Srm) plays an important role in polyamine synthesis. Under the conditions of liver injury, downregulation of Mat1a and upregulation of Mat2a are observed, resulting in a low level of AdoMet that has a protective effect against liver injury [SM6]. A database search demonstrated that Hsph1 interacts with Mat2a [SM7]. Pgs1 is responsible for the biosynthesis of phosphatidylglycerol and cardiolipin, located in the inner mitochondrial membrane, and the reactive oxygen species (ROS)-induced oxidation of these compounds is associated with mitochondrial dysfunction [SM8]. AdoMet is reported to prevent mitochondrial dysfunction triggered by chronic alcohol treatment [SM9].

Overall, the results indicate that the dysregulation of methionine metabolism and decrease in AdoMet are associated with hepatic necrosis, mitochondrial dysregulation, and cell growth caused by acetaminophen treatment. These reports are summarized in Figure SM3.

**Computational environment**

The AI Bridging Cloud Infrastructure (ABCI) operated at the National Institute of Advanced Industrial Science and Technology (AIST), Japan, was used for the experiments.

**Implementation**

Our method is implemented in Python 3.0 and is dependent on the *pandas*, *joblib*, and *os* modules. The detection of frequent itemsets was conducted by a modified *apriori* function in the *mlxtend* Python module, to introduce fuzzy logic.

**Protocol for reading findings of NLP**

***Tagging protocol***

To assign tags to a range of expressions that appear in clinical texts, such as consultation records and reading findings, which correspond to medical concepts such as names of diseases, disorders, and sites, tag classification was performed according to newly developed annotation guidelines

(URL:https://sociocom.naist.jp/real-mednlp/wp-content/uploads/sites/3/2021/12/Real-MedNLP_Annotation_Guidelines.pdf.).

***Explanation of the knowledge extraction model***

According to the tag classification described above, annotation guidelines were developed based on actual cases. The tagging and extraction of important words and phrases were performed using medical examination records and reading findings. The validity of the tagging was checked by experts (MD, PhD in Pharmacology, who are specialists in respiratory medicine) with medical knowledge, and correct data were generated. Using the obtained corpus, we set up a medical expression recognition and relationship estimation system and constructed an extraction system using the Japanese BERT model.

**MOFA2**

As the SB analysis, MOFA2 was applies to the three pairs, proteome data and CT interpretation report, proteome data and medical record, and proteome data and blood test, respectively, excluding the diagnosis categories, IIP and HC, in each dataset. A Welch's t-test was performed between samples belonging to each of the two categories for each Factor. If the p value was less than 0.05, the top 30 largest features sorted by the weight were selected for each dataset. The sample was divided into two groups for each Factor: the High group comprising the top 25% of individuals based on their factor values, and the Low group comprising the remaining individuals. The correlation　coefficients of features were calculated in the High group and the Low group, respectively, where Pearson correlation was used for continuous variables and point-biserial correlation was used for binary variables. Further features were selected in accordance with the selection criteria; the correlation coefficient of the High group was greater than 0.7, that of the Low group was smaller than or equal to 0.7, and difference between them was greater than 0.5. Proteins belonging to proteome dataset were extracted from the selected features.

**Similarity Network Fusion (SNF)**

SNF was applies to the three pairs, proteome data and CT interpretation report, proteome data and medical record, and proteome data and blood test, respectively, excluding the diagnosis categories, IIP and HC, in each dataset. Each dataset was standardized prior to analysis. To construct the integrated similarity networks, we performed a grid search to optimize the hyperparameters, with K (number of nearest neighbors) ranging from 10 to 60 and μ (scaling parameter) from 0.3 to 0.8. Sample affinities were calculated for each dataset and subsequently fused into a unified network. Finally, spectral clustering was used to partition the fused network into two groups, and the clustering performance was evaluated against the true diagnosis groups, IIP vs. HC, using Normalized Mutual Information (NMI) and Adjusted Rand Index (ARI).

**Visualization of the proteome data**

Proteomic data were log-transformed (base:10) before visualization. The heatmap was created with *seaborn* Python module (*29*) with the following parameter settings: method='average,' metric='cosine,' z_score=1, standard_scale=None. For t-SNE and UMAP, proteome data were further converted into z-scores. The t-SNE was conducted using the *scikit-learn* Python module (*30*) with the following parameter settings: n_components=2, perplexity=5, and metric='cosine.' UMAP was conducted using the *umap* Python module (*31*) with the following parameter settings: n_components=2, n_neighbors=5, and metric='cosine.'

**References for Supplementary Methods**

SM1 Bushel, P. R., Wolfinger, R. D. & Gibson, G. Simultaneous clustering of gene expression data with clinical chemistry and pathological evaluations reveals phenotypic prototypes. *BMC Syst. Biol.* **1**, 15 (2007).

SM2 Nassar, I., Pasupati, T., Judson, J. P. & Segarra, I. Histopathological study of the hepatic and renal toxicity associated with the co-administration of imatinib and acetaminophen in a preclinical mouse model. *Malays. J. Pathol.* **32**, 1-11 (2010).

SM3 Kishi, S. *et al.* Preexisting diabetes mellitus had no effect on the no-observed-adverse-effect-level of acetaminophen in rats. *J. Toxicol. Sci.* **45**, 151-162 (2020).

SM4 Martínez-Chantar, M. L. *et al.* Importance of a deficiency in S-adenosyl-L-methionine synthesis in the pathogenesis of liver injury. *Am. J. Clin. Nutr.* **76**, 1177S-1182S (2002).

SM5 James, L. P., Mayeux, P. R. & Hinson, J. A. Acetaminophen-induced hepatotoxicity. *Drug Metab. Dispos.* **31**, 1499-1506 (2003).

SM6 Lu, S. C. & Mato, J. M. S-adenosylmethionine in liver health, injury, and cancer. *Physiol. Rev.* **92**, 1515-1542 (2012).

SM7 Rouillard, A. D. *et al.* The harmonizome: A collection of processed datasets gathered to serve and mine knowledge about genes and proteins. *Database* **2016** (2016).

SM8 Paradies, G., Paradies, V., Ruggiero, F. M. & Petrosillo, G. Oxidative stress, cardiolipin and mitochondrial dysfunction in nonalcoholic fatty liver disease. *World J. Gastroenterol.* **20**, 14205 (2014).

SM9 Bailey, S. M. *et al.* S-adenosylmethionine prevents chronic alcohol-induced mitochondrial dysfunction in the rat liver. *Am. J. Physiol. Gastrointest. Liver Physiol.* **291**, G857-G867 (2006).

SM10 Dennis, G. *et al.* DAVID: database for annotation, visualization, and integrated discovery. *Genome Biol.* **4**, 1-11 (2003).

**Figures for Supplementary Methods**

Figure SM1. Generation of artificial data.

1. Artificial data (small). Two matrices with 100 rows and 200 columns, whose values follow standard normal distribution, were generated assuming that each row represents an observation (e.g., patient) and each column represents an attribute (e.g., a gene or a clinical measurement). Let one matrix be data1 and the other be data2 in Supplementary figure SM1a. For the first 10 rows, values in columns 1, 2, and 3 of data1 and values in 10, 20, and 30 of data2 were replaced by values that followed *N*(3, 0.5). For the second 10 rows, values in columns 20, 40, and 60 of data1 and values in 40, 80, and 120 of data2 were replaced by values that followed *N*(-3, 0.5). For the third 10 rows, values in columns 50, 75, and 100 of data1 and values in columns 50 and 100 of data2 were replaced by values that followed *N*(3, 0.5), and values in columns 125 and 150 of data1 and a value in column 150 of data2 were replaced by values that followed *N*(-3, 0.5). b) Artificial data (large). Two matrices with 1000 rows and 2000 columns, whose values follow standard normal distribution, were generated and certain values were replaced, as shown in Supplementary figure SM1b, using the same procedure as under “Artificial data (small)” in Supplementary figure SM1a.

Figure SM2. Summary of results of experiment with liver toxicity dataset

a) Liver toxicity data [SM1] used for the experiment. We applied our algorithm to this dataset to find genes (data1) that related to histopathological observations and/or clinical measurements (evaluation criteria for the degree of liver toxicity by administration of acetaminophen) and/or experimental conditions of administration of acetaminophen (data2). Data1 had 64 rows (rats) and 3116 columns (genes), and data2 had 64 rows (rats) and 60 (= 48+10+2) columns (48 histopathological observations, 10 clinical measurements, and 2 experimental conditions). b) Paired-association rule detected by this experiment. Among the 3986 paired-association rules detected (threshold: lift (frequent itemsets from data1 → frequent itemsets from data2) = 4.8), one rule with the highest conviction is shown in Supplementary figure SM2b. Ten genes were related to five clinical measurements, four histopathological observations, and two experimental conditions.

Figure SM3. Inferred molecular basis of liver injury by acetaminophen

Among the genes shown in Supplementary figure SM2b, 4 were found to be involved in liver injury. Their relationships and associated biological events are depicted. Hsph1: heat shock protein family H (Hsp110) member 1, Mat2a: methionine adenosyltransferase 2A, Pgs1: phosphatidylglycerophosphate synthase 1, Srm: spermidine synthase, GSH: glutathione.

**Tables for Supplementary Methods**
