# Supplementary material for: Subset binding enables detection of multimodal patient subgroup patterns and drug target discovery in idiopathic pulmonary fibrosis
Source: Brief Bioinform. 2026 Apr 14;27(2):bbag153. doi: 10.1093/bib/bbag153 (PMC13076932; doi:10.1093/bib/bbag153)
Supplement: Supplementary_material_bbag153 [file supplementary_material_bbag153.zip › List of Supplementary Materials.docx]

List of Supplementary Materials

Supplementary Methods Subset binding: A novel algorithm to detect paired itemsets from heterogeneous data including biological datasets

Supplementary Table 1 List of Items Derived from Clinical Information

Supplementary Table 2 Proteins Identified Through Serum Exosomal Proteomic Analysis

Supplementary Table 3 Twenty IPF-Related Proteins Identified Through Subset Binding

Supplementary Table 4 Proteins Identified by MOFA2

Supplementary Table 5 Phenotype of Knockout Mice for IPF-Related Proteins

Supplementary Table 6 Quantitative Evaluation of IPF-Related Protein Expression in Fibrotic and Normal Areas of Lungs from IPF Patients
